# Advances in Drug Delivery and Biomaterials: Facts and Vision

**DOI:** 10.3390/pharmaceutics11010048

**Published:** 2019-01-21

**Authors:** Paolo Caliceti, Pietro Matricardi

**Affiliations:** 1 Department of Pharmaceutical and Pharmacological Sciences, University of Padova, via Marzolo, 5 35131 Padova, Italy; paolo.caliceti@unipd.it; 2 Department of Drug Chemistry and Technologies, Sapienza University of Roma, P.le Aldo Moro 5, 00185 Roma, Italy

**Keywords:** drug delivery, biomaterials, controlled release

## Abstract

Drug delivery and biomaterials are different fields of science but, at the same time, are tightly related and intertwined. The 2018 CRS Italy Chapter Annual Workshop aims to explore recent advances in design and development in these areas. Many colleagues from Europe participated to the Workshop, stimulating the discussion. To foster the discussion on recent research and networking opportunities, especially among younger attendees, all poster-presenting authors were asked to provide a short talk. The very friendly and stimulating atmosphere allowed the attendees to explore new frontiers and tackle new horizons.

## 1. Aim and Scope of the Meeting

The 2018 Controlled Release Society (CRS) Italy Chapter Annual Workshop was held in Padova, 18–20 October 2018. The University of Padova kindly supported the Workshop, offering two wonderful venues: the new main conference room of the “Orto Botanico”, heritage of humanity (Botanical Garden, http://www.ortobotanicopd.it/en), and the main hall of the “Palazzo del Bo” (https://www.unipd.it/palazzo-bo) in Padua.

The workshop theme was Advances in Drug Delivery and Biomaterials: Facts and Vision. While drug delivery and biomaterials are different fields of science, they are, at the same time, tightly related and intertwined. The 2018 CRS Italy Chapter Annual Workshop aimed at exploring recent advances in design and development in these areas. A number of colleagues from all over Europe lectured and stimulated the discussion on exploring new frontiers and tackling new horizons. To foster discussion on recent research and networking opportunities among attendees, all poster presenters were provided with the opportunity to give a short talk (10 min, in front of the poster).

Over 100 delegates from universities and pharmaceutical companies, mainly from Italy, attended the workshop, which featured 14 speakers with diverse research interests and backgrounds. A brief summary of the presentations of the invited speakers and the abstracts of the posters are reported hereafter.

## 2. Lectures

### 2.1. Adaptive Nanoparticles for Nanomedicine Applications

van HestJan C.M.Radboud University, Bio-Organic Chemistry Heyendaalseweg 135, 6525 AJ Nijmegen, The Netherlands; j.vanhest@science.ru.nl

Polymer vesicles, or polymersomes, are highly versatile carrier systems which have found widespread application in the area of nanomedicine. In most cases, polymersomes are used as closed spherical containers that effectively transport their cargo to the desired site of action in the human body. However, for certain applications, nanoparticles are required with adaptive features and unusual topologies. In this presentation, three different topics will be discussed. Enzyme replacement therapy is an efficient method to treat a number of metabolic diseases which are the result of dysfunction of one enzyme. However, current therapies are expensive, as the enzymes have only a short period of activity. We have developed a biodegradable polymersome nanoreactor which comprises and protects enzymes. The nanoreactor is semipermeable, as it allows the passage of only small-molecule products and substrates. The polymersomes were shown to be active as artificial organelles in patient cells to treat oxidative stress.

Non-spherical-shaped polymersomes can be of importance in applications such as vaccine development, in which the interaction of immune cells with the antigen carriers is strongly dependent on shape. One of the most intriguing morphologies other than spheres are tubular structures, as they show resemblance to bacterial topologies, and would provide a larger contact surface area between particles and cells. We have developed a number of methodologies that allow us to reshape spherical vesicles into tubular ones. The ability to functionalize the particle surface makes these structures amenable for application in the immunology field.

A final example of an adaptive nanoparticle was created by the layer-by-layer technique. One of the layers was composed of the anti-thrombolysis compound, heparin. The LbL capsules were partially covered with a gold shell, which provided them, upon irradiation with NIR light, with autonomous movement. Upon increasing the NIR laser intensity, the capsules were disassembled and the heparin was released. This specific behavior was used to steer the particles to thrombus plaques, which were subsequently dissolved.

### 2.2. Melanin Binding: Approach to Targeted Drug Delivery?

UrttiArtoUniversity of Helsinki, University of Eastern Finland and St Petersburg State University, P.O. Box 33 (Yliopistonkatu 4), 00014 Helsinki, Finland; arto.urtti@helsinki.fi

Melanin binding of small molecular drugs has been known for decades, but the chemical drivers and cellular pharmacokinetics related to melanin binding are unknown. We explored the binding of compounds to melanin and established the following: (1) melanin binding is relatively non-specific and has many different binding energies; (2) binding could be predicted on chemical structure at 90% accuracy based on a machine learning based model; (3) cellular pharmacokinetics depends strongly both on the binding and membrane permeability of the drug in plasma membranes and melanosomal membranes. This work helps the rational design of compounds that home in on melanin, and thus, could show targeted and prolonged drug exposure in the eye tissues, such as retina and choroid.

### 2.3. Nanomedicine in Cancer: Toward Overcoming Chemoresistance

CampaniVirginiaGiarraSimonaDe RosaGiuseppe*Department of Pharmacy, Federico II University of Naples, Via Domenico Montesano, 49, 80131 Napoli, Italy*Correspondence: gderosa@unina.it

Cancer represents one of the diseases with a major impact on society across the world. In spite of significant advances in understanding the etiology and progression of cancer, and in developing novel diagnostics and therapeutics, both the incidence and the mortality rates of malignancies remain extremely high. Intrinsic and acquired drug resistance (multidrug resistance or MDR) is a major challenge in treating cancer patients, leading to cancer recurrence, dissemination and death. One of the most frequent causes of chemoresistance is the elevated expression levels of drug efflux pumps, such as P-glycoprotein (Pgp) belonging to the ATP-binding cassette (ABC) superfamily.

Nanomedicine represents a promising tool to overcome MDR at different levels. First of all, nanovectors have been proposed to deliver ABC inhibitors co-administered or co-encapsulated with chemotherapeutics. Molecules with a well-established activity of ABC-inhibitors, such as verapamil, have shown systemic toxicity, while alternative strategies to inhibit ABC expression are emerging e.g., with the bisphosphonates as zoledronic acids (ZOL). The latter is able to inhibit the mevalonate pathway that has been correlated with the overexpression of ABC in MDR cells with a poor clinical outcome. However, the rapid accumulation into bone limits the clinical use of ZOL to treat diseases located in extraskeletal tissues. Recently, we demonstrated that the use of nanovectors such as liposomes and self-assembling nanoparticles (SANPs) can be used to deliver ZOL in tumors. We also observed that ZOL, only by using nanovectors, reduces the expression of Pgp in MDR cancer cells, thus restoring the sensitivity of MDR tumors to chemotherapeutics such as doxorubicin or carboplatin. More recently, chitosan-based nanoparticles have been developed to co-encapsulate ZOL and doxorubicin for combined therapy against MDR tumors. In this case, a synergistic inhibition activity was also observed when combining ZOL and doxorubicin on MDR cells. A second level in which the nanomedicine can be used to overcome MDR is the delivery of nucleic acids such as small interfering RNA (siRNA) or microRNA (miRNA). Indeed, siRNAs can be used to inhibit the expression of a protein involved in MDR, while miRNAs are important modulators or protein expression, and their mis-expression has been correlated with the occurrence of MDR. Thus, polymeric micelles have developed for the co-delivery of an anti-survivin siRNA and paclitaxel for the reversal of drug resistance in tumors. On the other hand, SANPs encapsulating the miR603 have been proposed to restore sensitivity to temozolomide in glioblastoma cells (unpublished data). Finally, A third level of attention should be paid to the biomaterials used when using nanovectors in MDR tumors. The use of liposomes can overcome resistance to doxorubicin in cells overexpressing the Pgp. In addition, the use of suitable biomaterials, and in particular the inclusion of PEGylated lipid in the formulation, can be particularly useful to inhibit the activity of the efflux pumps.

**Acknowledgments:** This work has been partially supported the Italian Ministry of Education, University and Research (MIUR) with a project (FIRB-ACCORDI DI PROGRAMMA 2011 and PRIN 2009) and by Phospolipid Reserch Center of Heidelberg.

### 2.4. Exploring Nano- and Micromaterials to Combat Infectious Diseases. About Our Failures and Successes

De SmedtS.C.*BraeckmansK.DemeesterJ.Department of Pharmaceutics of Ghent University, Ghent Research Group on Nanomedicines, Biophotonic Research Group, Ottergemsesteenweg 460, B-9000 Ghent, Belgium*Correspondence: Stefaan.DeSmedt@UGent.be

The implementation of prophylactic vaccines in modern medicine turned out to be an efficient weapon against infectious diseases. However, traditional vaccines consisting of live-attenuated or inactivated microorganisms raise considerable safety concerns shifting the trend in vaccine development towards the use of clearly-defined subunit proteins and peptides. Despite their improved safety profile, they often suffer low immunogenicity, hampering the induction of appropriate immune responses. Particulate vaccine delivery systems are promising candidates to overcome this barrier because particles can target antigen delivery to antigen-presenting cells, and are believed to be superior in eliciting an immune response compared to antigens alone. The first part of this lecture will summarize the successes and failures we experienced the last 10 years in our projects on polyelectrolyte (PE) microcapsules (designed by Layer-by-Layer coating) for vaccination purposes. In the second part of this lecture, we will present our recent findings on the improved killing of bacteria in biofilms by the combined use of antibiotics and nanomaterials. Indeed, efficient eradication of bacteria growing in biofilms remains a huge challenge; the reasons for this include the degradation of the antimicrobial agent before it reaches its target location, binding of the antimicrobial agent to non-target materials and the increased antimicrobial tolerance of biofilm bacteria. Nanotechnology-based concepts may become useful to supress the growth of biofilms and bacteria.

### 2.5. Nanoparticles for Drug Targeting: Current Status and Future

StormGert^1^,^2^1Dept. Pharmaceutics, Utrecht Institute for Pharmaceutical Sciences (UIPS), Utrecht University, PO Box 80082, 3508 TB Utrecht, The Netherlands; G.Storm@uu.nl2Dept. Biomaterials Science & Technology (BST), MIRA Institute for Biomedical Technology and Technical Medicine, University of Twente, 7522 NB Enschede, The Netherlands

One most active sectors of research within the field of nanomedicine has been the design of nanoparticulate pharmaceuticals for targeted drug delivery. In fact, novel and established nanoparticle systems continue to flourish in research laboratories. However, the number of such systems that have been approved for the treatment of patients is still limited. Examples are Caelyx/Doxil (doxorubicin), Myocet (doxorubicin), DaunoXome (daunorubicin), Marqibo (vincristine), Onyvide (irinotecan), Onco-TCS (vincristine), Vyxeos (cytarabine and daunorubicin) and Abraxane (paclitaxel). While these examples illustrate that significant advances have been made over the years in making nanoparticulate nanomedicines a clinical reality, there is nevertheless growing skepticism in the scientific literature regarding the future and clinical applicability of such targeted nanopharmaceuticals. In this presentation, I will discuss the arguments raised to justify this negative attitude, as well as my different view on the current status and future of the use of nanoparticles for drug targeting.

### 2.6. Nanotechnological Approaches to Enhance Anticancer Chemo-Immunotherapy

LolloGiovanna^1^,^2^*MarigoIlaria^3^BronteVincenzo^3^BenoitJean Pierre^2^1Univ Lyon, Université Claude Bernard Lyon 1, CNRS, LAGEP UMR 5007, 43 boulevard du 11 novembre 1918, F-69100 Villeurbanne, France2MINT, Université d’Angers, INSERM U1066, CNRS UMR 6021, F-49933 Angers, France3Section of Oncology and Immunology, Department of Surgery, Oncology and Gastroenterology, University of Padova, 35128 Padova, Italy*Correspondence: giovanna.lollo@univ-lyon1.fr

Over the last decade, great efforts have been dedicated to the development of anticancer immunotherapies that are able to induce or boost an existing immune response against neoplastic cells [1]. In this work, a double therapeutic approach that combines the depletion of circulating myeloid-derived suppressor cells (MDSCs) and the stimulation of a specific immune response against tumors is presented. Firstly, we revert immunosuppression by targeting MDSCs through the use of drug-loaded nanosystems. Then, we trigger a tumor-specific immune response by inducing anticancer adoptive T cell therapy (ACT). Monocytic-MDSC (M-MDSC) are immunosuppressive myeloid cells known to impair the efficacy of cancer immunotherapy while promoting neovascularization and metastasis formation assisting cancer cells [2]. Lipid nanocapsules (LNCs) loaded with a lauroyl-modified form of gemcitabine (GemC12) efficiently target the M-MDSC subset in vitro. Moreover, in vivo studies performed following the subcutaneous administration of GemC12-loaded LNCs reduced the percentage of spleen and tumor-infiltrating M-MDSCs in lymphoma and melanoma-bearing mice, with enhanced efficacy when compared to free gemcitabine. Based on these results, we evaluated the therapeutic efficacy of an activate T cell transfert (ACT) protocol, in which GemC12-loaded LNCs were administered as preconditioning treatment. GemC12-LNCs significantly increased the efficacy of ACT using OVA and TERT-specific T cells, indicating enhanced treatment potency associated with drug encapsulation [3]. Then, in order to develop a combined nanotechnological approach, a strategy based on direct stimulation of tumor cell death (ICD), novel polymeric nanoparticles loaded with an oxaliplatin derivatives (DACHPt) were obtained. Oxaliplatin, an ICD inducer, was efficiently encapsulated into stable polymeric biodegradable nanoparticles [4]. Following a rational optimization of the system design, nanoparticles demonstrated the ability to induce HGMB1 and ATP release, which are two major events related with ICD. Moreover, the intravenous administration of DACHPt-nanoparticles can ensure a higher drug exposure over a prolonged time without drug accumulation. Further works will be carried out to exploit the combinatorial nanoparticulate-chemotherapeutic approach to obtain a synergistic and long-lasting approach in anticancer immunotherapy.

**References:** [1] Sengupta, S. Cancer Nanomedicine: Lessons for Immuno-Oncology. *Trends Cancer* **2017**, *3*, 551–560. [2] Ugel, S.; Peranzoni, E.; Desantis, G.; Chioda, M.; Walter, S.; Weinschenk, T.; Ochando, J.C.; Cabrelle, A.; Mandruzzato, S.; Bronte, V. Immune tolerance to tumor antigens occurs in a specialized environment of the spleen. *Cell Rep.* **2012**, *2*, 628–639. [3] Sasso, M.S.; Lollo, G.; Pitorre, M.; Solito, S.; Pinton, L.; Valpione, S.; Bastiat, G.; Mandruzzato, S.; Bronte, V.; Marigo, I.; et al. Low dose gemcitabine-loaded lipid nanocapsules target monocytic myeloid-derived suppressor cells and potentiate cancer immunotherapy. *Biomaterials* **2016**, *96*, 47–62. [4] Lollo, G.; Benoit, J.P.; Brachet, M. Drug Delivery System European. Patent EP18306201.7, 22 June 2018.

**Acknowledgments:** This work was supported by EuroNanoMed II 2013 (NICHE); EuroNanomed 2009 (Lymphotarg); Italian Association for CancerResearch (AIRC, grants IG 14103 and IG 12886); FIRB (cup:B31J110004200010).

### 2.7. Ophthalmic Protein Drug Packaging: Critical Aspects, Existing Solutions and Opportunities. A Case Study

GentileMarco M.Dompé Farmaceutici S.p.A., Via Campo di Pile, 67100 L’Aquila, Italy; marco.gentile@dompe.com

Topical eye drops are the most convenient and patient compliant route of drug administration, especially for the treatment of anterior segment diseases. More than 70% of ophthalmic drug products are simple solutions supplied in multi-dose plastic container closure systems (CCS), which generally contain a weekly or longer supply of the drug. These products may be intended for the treatment of acute or chronic conditions. In the 1950’s, the introduction of the use of preservatives in ophthalmic products to prevent contamination after opening constituted a considerable advance, allowing the use of multidose containers. Thirty years later, numerous publications have shown the unfavorable effects of preservatives on the cornea, the conjunctiva and the tear film, causing irritation, inflammation and dry eye. In order to avoid this problem, single sterile dose units were put on the market (i.e., blow, fill seal (BFS) technology). From the 1990’s, also multi-dose bottles (Figure 1) capable of dispensing preservative-free eye drops (PFMD) appeared on the market: the first was ABAK^®^ system from Thea, followed by a series of preservative-free packaging forms such as OSD^®^ Aptar and Novelia^®^ Nemera technologies.

Almost all ophthalmic drug products currently on the market contain “small molecule” drug substances; protein drugs are very rare. Furthermore, protein drugs are conventionally packaged (as solutions or freeze-dried powders to be reconstituted) in glass containers. Hence, the development of plastic primary packaging (i.e., the most friendly and practical systems to administer eye drops) for an ophthalmic protein drug product represents a critical but challenging research opportunity. The compatibility of a protein formulation with its primary packaging and container closure system is key to maintaining the stability of the drug product and to preserving its safety and efficacy. Incompatibility can occur by not fully understanding the material surface properties of the container. The key consideration for initial container selection should be to choose components that maintain product stability by minimizing protein adsorption, extractables/leachables, oxidation, and pH changes.

**Figure 2:**
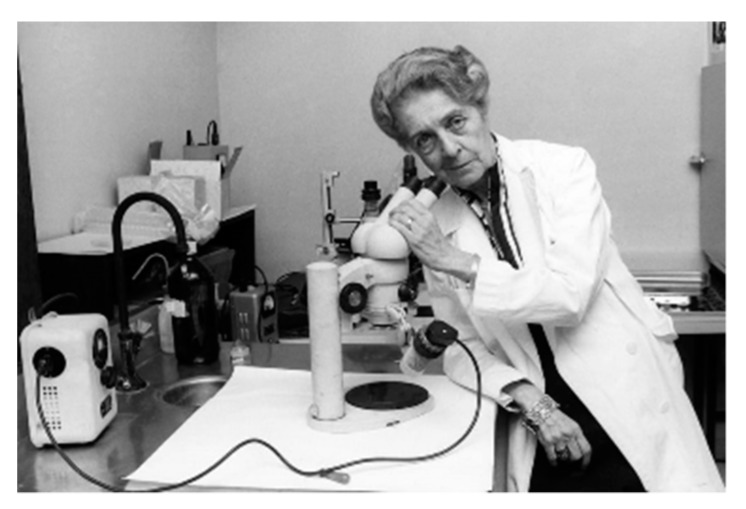
Rita Levi Montalcini (Nobel Prize in 1986 for Nerve Growth Factor discovery).

OXERVATE^®^ case study. Oxervate (brand name of cenegermin eye drops solution) has been recently approved in Europe and US with indication in moderate and severe neurotrophic keratitis (NK). Cenegermin is the INN name of rh-Nerve Growth Factor (rhNGF), a protein (neurotophin) discovered by Rita Levi Montalcini (Figure 2). NGF is a naturally occurring protein in humans involved in differentiation, growth, maintenance and survival of neurons.

The therapeutic potential of NGF in the treatment of neurotrophic keratitis had been proposed in the late 90s, and was supported by preliminary results from open-label clinical studies with murin NGF extracted from mice sub-maxillary glands. In 2010, Dompé acquired the rights for rhNGF development, manufacturing and commercialization in ophthalmology (acquisition of Anabasis) and started new development studies. Consequently, human, nonimmunogenic, recombinant NGF (rhNGF) became available for the clinical treatment of eye neuropathies in humans.

Clinical indications. Neuropathies may affect both the anterior and the posterior segments of the eye, causing either an epithelial defect or an interruption in the transmission of visual information between retina and brain (i.e., optic nerve neuropathies). Neurotrophic keratitis (NK), a corneal debilitating and progressive pathology, was chosen as the first clinical indication among other eye pathologies to be treated potentially with rhNGF.

Evidence of clinical efficacy. The healing of the cornea obtained after rhNGF treatment of patients with persistent epithelial defects unresponsive to lubricant, therapeutic contact lenses and amniotic membrane transplants was achieved in May 2013 at Moorefield H.—UK (Prof. J. Dart). Clinical safety and efficacy studies in volunteers and patients with neurotrophic keratitis (NK) at stage 2 and 3 were planned and concluded in the period 2013–2015. Oxervate eye drops have been shown to be efficacious and to have a good safety profile. The improvement of all symptoms was assessed by VAS score and complete healing in >70% patients associated with improvement in sensitivity at week 8 were obtained.

Drug product formulation. The development of the formulation was addressed to obtain a stable rhNGF solution with suitable characteristics for ocular instillation (pH and osmolality in particular). Excipients already used in ophthalmic preparation were preferred. The stability of rhNGF formulated solutions at different concentrations was investigated in the range of temperature −20 °C–+25 °C. According to the obtained results, no deamidation was observed; only an oxidized rhNGF form was observed in some cases in the formulations after 3–9 months of storage (i.e., rh NGF contains two methionyl residues which are potential sites of oxidation). Chemical oxidative instability was minimized by the appropriate choice of preparation procedures, lowering O_2_ head space, using inertized containers and by the addition of an antioxidant. Consequently, methionine as component of formulation, O_2_ containment during the preparation and O_2_ head space reduction after filling and before sealing of the container were introduced.

**Figure 3:**
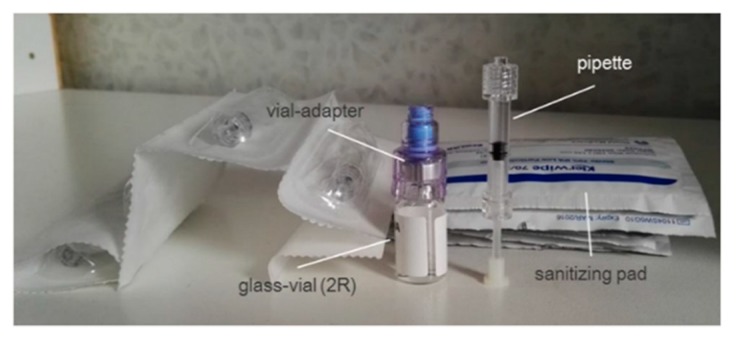
Oxervate^®^ administration kit.

Current drug product primary packaging. The product is currently packaged into a siliconized clear glass class-I vial closed with a rubber stopper and a flip-off aluminum cap (Figure 3). Each vial contains 1.0 mL of the product. The ocular administration of the drug product is achieved by means of a plastic vial adapter positioned on the stopper head to which a polycarbonate pipette (with a capacity of 120 mcl) is connected in order to withdraw the solution and deliver a drop of liquid into the eye. Six administrations per day from a single vial and using 6 pipettes are carried out (daily multidose vial).

New primary packaging studies. The current standard for ophthalmic medications is unpreserved sterile Blow Fill Seal (BFS) single dose or preservative-free sterile multi-dose (PFMD). For chronic eye care treatment, multi-dose systems seem to be the most convenient and cost effective. Primary packaging (i.e., bottles) is made of pharma grade plastics, mainly LDPE, HDPE and various copolymer between PE and PP or COC. For rhNGF protein drug, the major issues in developing and selecting a plastic container are oxidation (from leachables, O_2_) and adsorption to surfaces. Hence, along with conventional stability studies, investigation of the mechanisms of interaction between rhNGF protein and plastic materials, and of the influence of the surface characteristics on the absorption kinetic along the drug product shelf life has been carried out. Two major techniques have been used: Atomic Force Microscopy and Crystal quartz microbalance with dissipation monitoring (QCMD). With the Atomic Force Microscopy’s cantilever, we have scanned the surface of material in order to investigate the surface roughness before and after contact with the protein solution. QCM-D can measure, in quantitative terms, the total amount of protein deposited on the material over time. It is a very sensitive technique: mass sensitivity in air about 1 ng/cm^2^, in liquid about 5 ng/cm^2^. Hydrophobic, hydrophilic and wettability characteristics of the materials have been investigated with contact angle analysis between the material vs. several rhNGF protein formulations.

### 2.8. Bile Acids: New Opportunities for the Drug Targeting?

DalpiazAlessandroDepartment of Chemical and Pharmaceutical Sciences, University of Ferrara, via Fossato di Mortara 19, I-44121 Ferrara, Italy; dla@unife.it

Currently, nanoparticulate systems appear promising to potentiate the therapeutic effects of drugs that, upon administration in free form, can have great difficulties in reaching target sites in the body, such as HIV “sanctuaries”, or cancer cells showing multidrug resistance (MDR). The modulation of the uptake of nanoparticles by macrophages appears to be relevant in this regard. Indeed, an efficacious macrophage uptake is necessary in order to eradicate intracellular pathogens, such us HIV in brain macrophages, considered as a sanctuary for this virus. The lack of penetration of anti-HIV drugs in the macrophages is attributed to the expression of active efflux transporters (AET) on their membranes. The nanoparticulate systems, able to elude AETs, can constitute an excellent vehicle which is able to target their encapsulated drugs in macrophages; their ability to ingest and disrupt the foreign material in the body is well known. On the other hand, when the uptake activity is exerted by the macrophages of the reticuloendothelial system (RES), the nanoparticles are easily and very quickly removed from the bloodstream before they can perform their designed therapeutic function, such as a selective targeting of anticancer drugs in tumoral tissues or cells, by passive or active mechanisms. In order to obtain nanocarriers with stealth properties against the RES, polyethylene glycol (PEG) is currently used as a coating material for the surfaces of nanoparticles, even if it can induce immune reactions and body accumulation risks [1].

Formulations able both to elude the AET systems and to modulate their uptake by macrophage may therefore be of great utility in order to target therapeutic sites. Considerable evidence suggests that an appropriate use of bile acids may be very useful in this sense. For this type of study, we chose Zidovudine (AZT) as the model drug, being a nucleoside reverse transcriptase inhibitor currently used in highly-active antiretroviral therapy against HIV and substrate of AETs expressed by the blood brain barrier and macrophages, and which is therefore unable to reach HIV sanctuaries in the central nervous system (CNS). Its conjugation with a bile acid (ursodeoxycholic acid, UDCA) allowed us to obtain a lipophilic prodrug (UDCA-AZT) which is able to elude the AET systems of which it is a substrate [2], with a consequent increase of the uptake in murine macrophages [3]. This prodrug, in contrast to AZT, can easily be encapsulated in polymeric or solid lipid microparticles used as nasal formulations in order to induce the prodrug uptake and a prolonged permanence in the cerebrospinal fluid (CSF) [3,4], where it can permeate in the CNS macrophages.

Very recently, we have shown that nanoparticles obtained by nanoprecipitation of UDCA-AZT in the presence of taurocholate or ursodeoxycholate are strongly or weakly taken up by murine macrophages, respectively. This new and unexpected result opens new perspectives in easily obtaining stealth or poorly-taken up nanoparticulate systems. The core of these nanoparticles, surrounded by a bile acid coating, can be constituted solely of a lipophilic drug or prodrug, evidencing high biocompatibility and simple and cheap formulation procedures.

**References:** [1] Wang, M.; Thanou, M. Targeting nanoparticles to cancer. *Pharmacol. Res.* **2010**, *62*, 90–99. [2] Dalpiaz, A.; Paganetto, G.; Pavan, B.; Fogagnolo, M.; Medici, A.; Beggiat, S.; Perrone, D. Zidovudine and Ursodeoxycholic Acid Conjugation: Design of a New Prodrug Potentially Able to Bypass the Active Efflux Transport Systems of the Central Nervous System. *Mol. Pharm.* **2012**, *9*, 957–968. [3] Dalpiaz, A.; Fogagnolo, M.; Ferraro, L.; Capuzzo, A.; Pavan, B.; Rassu, G.; Salis, A.; Giunchedi, P.; Gavini, E. Nasal chitosan microparticles target a zidovudine prodrug to brain HIV sanctuaries *Antivir. Res.* **2015**, *123*, 146–157. [4] Dalpiaz, A.; Ferraro, L.; Perrone, D.; Leo, E.; Iannuccelli, V.; Pavan, B.; Paganetto, G.; Beggiat, S.; Scalia, S. Brain uptake of a Zidovudine prodrug after nasal administration of solid lipid microparticles. *Mol. Pharm.* **2014**, *11*, 1550–1561.

### 2.9. Repurposing Cationic Amphiphilic Molecules to Promote Cellular Delivery of Therapeutics

KoenRaemdonckLaboratory for General Biochemistry and Physical Pharmacy, Ghent University, Ottergemsesteenweg 460, 9000 Gent, Belgium; koen.raemdonck@UGent.be

Small interfering RNAs (siRNAs) are attractive therapeutics to reduce the expression of disease-related genes, outperforming traditional small molecule drugs in terms of design, selectivity, and their ability to silence targets previously regarded as ‘undruggable’. To be functional, siRNAs require delivery into the cell cytosol. However, RNAs do not have optimal drug-like properties, as they lack the ability to cross biological membranes. To overcome extra-and intracellular barriers, RNA drugs are typically formulated into polymer- or lipid-based nanoparticles (i.e., nanomedicines). In recent years, many (pre-)clinical trials involving siRNA nanomedicines have demonstrated promising results, but have also identified many remaining hurdles that limit broad clinical translation. From a cellular delivery perspective, nanomedicines can guide macromolecules like siRNAs into cells through endocytosis; however, escape from the endosomal lumen into the cytosol prior to lysosomal degradation remains a major obstacle towards efficient intracellular drug delivery. Despite decades of research, even for current, state-of-the-art nanocarriers such as siRNA-loaded lipid nanoparticles, endosomal escape is a very inefficient process, with only ~1% of the internalized dose reaching the cell cytosol. This presentation will describe the repurposing of two distinct cationic amphiphiles, i.e., both low molecular weight cationic amphiphilic drugs (CADs), as well as the lung-related surfactant protein B (SP-B), to improve cellular delivery of small RNA therapeutics. Both approaches significantly promote cytosolic siRNA delivery efficiency, albeit by adopting a different mode-of-action.

### 2.10. Drug Carrier Transport across the Blood-Brain Barrier: An Elusive Parametric Balance

MuroSilvia^1^,^2^1Fischell Department of Bioengineering and Institute for Bioscience and Biotechnology Research, University of Maryland, College Park, 4500 Knox Road, MD 20740, USA; smuro@ibecbarcelona.eu2Institució Catalana de Reserca i Studis Avançats and Institute for Bioengineering of Catalonia of the Barcelona Institute of Science and Technology, Baldiri Reixac, 10-12 | 08028 Barcelona, Spain

Accessing the brain is key to studying its function and pathology, and for diagnostic and therapeutic purposes. Yet, this remains a great challenge due to the blood-brain barrier (BBB). To overcome this, novel nanovehicles are being designed to cross this interface, without much translational success. A prime obstacle is the lack of knowledge of the “biological regulation” of these devices, as most efforts have been devoted to controlling their chemical and physical properties, otherwise necessary tasks. Research in our group is focused on bridging this gap of knowledge. For this purpose, we have designed model nanovehicles targeted to receptors of the main routes of transcytosis across endothelial barriers (clathrin, caveolae, and cell adhesion molecule -CAM- identified byour lab), to compare their properties and BBB transport ability in cellular and animal models, using fluorescent and radioactive tracers. Engagement of receptors of the three routes by drug nanocarriers coated with targeting antibodies resulted in vesicular transport across the endothelial lining. The CAM pathway, in contrast to clathrin and caveolar routes, was effective across a broad spectrum of carrier sizes and targeting valencies. This is reminiscent of the CAM function, which contributes to transcellular leukocyte migration. We observed that this is because the CAM route associates with a precise remodeling of the lipid composition of the endothelial plasmalemma and reorganization of the actin cytoskeleton. Surprisingly, biophysical parameters of the drug carrier which improve binding and uptake by the endothelium do not always result in more efficient transcytosis, where an elusive balance must be met in order to optimize transport. As a result of said optimization, therapeutic cargoes such as enzyme for inherited neurodegenerative conditions were delivered to the brain in an active form after intravenous administration in mouse models. Improved delivery of therapeutics across the BBB in vivo illustrates the potential of nanodevices addressed to transcytosis routes as translational tools to improve CNS treatment.

### 2.11. Biotech Drugs Advances by Polymer Conjugation

PasutGianfrancoPharmaceutical and Pharmacological Sciences Department, University of Padova, 35131 Padova, Italy; gianfranco.pasut@unipd.it

Polymer covalent conjugation, especially with polyethylene glycol (PEG), is a consolidated strategy for improving the therapeutic performance of bioactive substances, like proteins, peptides, small drugs and oligonucleotides. Furthermore, this approach is playing an important role in introducing biocompatibility and increased in vivo half-life of other drug delivery systems such as liposomes, nanoparticles, nanotubes, etc. In general, polymer conjugation is performed to prolong the pharmacokinetic of a fast body-cleared molecule and to reduce immunogenicity. The former advantage is reached by decreasing the rates of both kidney clearance and degradation, while the latter is achieved by a shielding effect of the polymer’s chains over the immunogenic sites of a protein. So far, the polymer conjugation to protein was obtained by few chemical strategies, thus limiting the possibility to direct the polymer coupling to a desired site in view to minimize the activity lost.

Now this field is being renewed by taking advantage of the use of enzymes to mediate the polymer coupling to new sites in a protein, opening the possibility to obtain site-selective protein conjugates also in the case of very complex and high molecular weight proteins. In general, enzymatic conjugation is very specific for a predetermined site in a protein, and the conjugate formation is fast and often quantitative also in physiological conditions of reaction buffers.

Among the several enzymes introduced for PEGylation, this presentation will focus especially on the use of microbial transglutaminase.

**Acknowledgments:** This work was supported by AIRC (IG2017, Cod. 20224), University of Padova (STARS-WiC) and Italian Ministry of Health (“Ricerca Finalizzata” GR-2011-02351128).

### 2.12. Flower-Like and Golden Thermosensitive Micelles Using Native Chemical Ligation for Drug Delivery

NajafiMarziehHebelsErik R.HemburyMathewVermondenTina*Department of Pharmaceutics, Utrecht Institute for Pharmaceutical Sciences, Faculty of Science, Science for Life, Utrecht University, Universiteitsweg 99, 3584 CG Utrecht, The Netherlands*Correspondence: t.vermonden@uu.nl

Thermosensitive polymeric micelles are attractive as drug delivery vehicles because of their reversible self-assembling nature according totemperature. However, covalent crosslinking of micelles is considered to be of high importance to guarantee the stability of micelles in-vivo. Here, we introduced native chemical ligation (NCL) as a novel and straightforward method for covalent crosslinking of micelles in aqueous solution. This is an appealing method to covalently cross-link polymers because of its ability to react under physiological conditions, avoiding the use of toxic reagents and catalysts [1]. This specific ligation requires *N*-terminal cysteine and thioester functionalities, which also enable conjugation of desirable molecules using either amine or thiol moieties present in the polymers. In this study, NCL core-crosslinked thermosensitive flower-like micelles and star-like gold nanocluster containing micelles were investigated for drug delivery applications.

ABA triblock and AB diblock copolymers have been prepared by atom transfer radical polymerization (ATRP). Polyethylene glycol (PEG) was used as B-block and the A-blocks consisted of thermosensitive polyisopropylacrylamide (PNIPAM) decorated with either cysteine P(NIPAM-co-HPMA-Cys) (PNC) or thioester P(NIPAM-co-HPMA-ETSA) (PNE) functionalities, respectively. Aqueous solutions of these complementary polymers were mixed at 4 °C and rapidly heated to form either flower-like (ABA) or star-like (AB) micelles. Subsequently, native chemical ligation in the core of the micelles resulted in the stabilization of the micelles. Flower-like micelles displayed an average diameter of 65 nm at 37 °C. Changes in temperature between 10 and 37 °C only affected the size of the micelles by reversible collapse of the thermosensitive blocks. The polydispersity index (PDI) and aggregation number (*N_agg_*) were hardly affected by temperature changes, verifying the covalent stabilization of the micelles by NCL. Cryo-TEM and SLS measurements confirmed the formation of uniform and spherical micelles. Notably, by simply adjusting the molar ratio between the polymers, the extra cysteine or thioester moieties could be used for conjugation of functional molecules. In vitro cell experiments demonstrated that fluorescently-labeled micelles were successfully taken up by HeLa cells, while cell viability remained high, even at high micelle concentrations [2].

Analogous star-like micelles were prepared from AB block copolymers having an excess of thiol functionalities, which were used to link gold nanoclusters [3] associated with thiolated doxorubicin. Upon irradiation with a near infrared laser, this formulation showed a highly localized killing capacity in vitro using MDA-MB-231 breast cancer cells.

Concluding, Native Chemical Ligation as a biofriendly crosslinking method has shown itself to be very versatile for stabilizing micelles and as a linking strategy for fluorescent labels, gold nanoclusters and drugs.

**Acknowledgments:** The Netherlands Organization for Scientific Research (NWO/VIDI 13457 and NWO/Aspasia 015.009.038) is acknowledged for funding.

**References:** [1] Boere, K.W.M.; Soliman, B.G.; Rijkers, D.T.S.; Hennink, W.E.; Vermonden, T. Thermoresponsive injectable hydrogels cross-linked by native chemical ligation. *Macromolecules* **2014**, *47*, 2430–2438. [2] Najafi, M.; Kordalivand, N.; Moradi, M.; van den Dikkenberg, J.; Fokkink, R.; Friedrich, H.; Sommerdijk, N.A.J.M.; Hembury, M.; Vermonden, T. Native chemical ligation for cross-linking of flower-like micelles. *Biomacromolecules* **2018**, *19*, 3766–3775. [3] Hembury, M.; Beztsinna, N.; Asadi, H.; van den Dikkenberg, J.B.; Meeldijk, J.D.; Hennink, W.E.; Vermonden, T. Luminescent Gold Nanocluster-Decorated Polymeric Hybrid Particles with Assembly-Induced Emission. *Biomacromolecules* **2018**, *19*, 2841–2848.

### 2.13. Tailor-Made Functionalized Polymers for Nanomedicine

CavallaroGennaraLaboratory of Biocompatible Polymers, Dipartimento di Scienze e Tecnologie Biologiche Chimiche e Farmaceutiche (STEBICEF), University of Palermo, 90128 Palermo, Italy

Natural and synthetic polymers constitute the starting materials for the production of functionalized biomaterials able to produce nanoscale smart delivery systems very promising in nanomedicine applications. In this context chemistry has enormously contributed to the development of smart materials with specific bio-properties or able to be responsive for biomedical applications; besides the cooperation among biology, chemistry, chemical engineering, medicine made possible the combination of materials and architectures in order to generate new effective therapeutic nanosystems. The expertise of the Laboratory of Biocompatible Polymers concerns the design, synthesis and characterization of new functionalized polymers starting on either natural or synthetic polyaminoacids and polysaccharides to produce nanostructured drug delivery systems and nanomedicine useful for the treatment of different pathologies using different kinds of drugs including genetic materials. A wide number of pathologies represents still now unmet medical needs and for that, the use of nanotechnologies could represent an efficient solution. One of these pathologies is Cystic Fibrosis (CF). CF is an autosomal recessive disorder caused by mutations of the gene encoding the CF transmembrane conductance receptor (CFTR) which coding for a cAMP-dependent chloride channel protein. Cystic fibrosis leads to pathological changes in organs that express CFTR, including secretory cells, lungs, pancreas, liver, and reproductive tract but the progressive degeneration of pulmonary functionality is the main cause of death in CF patients. The CFTR gene codifies for a channel protein that allows the secretion of chloride ions. In patients with CF this protein is absent or there is a dramatic reduction of the functioning protein. The reduced secretion of chloride causes an uncontrolled reabsorption of sodium and water, inducing an important dehydration of mucus secretions. Mucus becomes so dense and viscous leading to the collapse of cilia and the subsequent inhibition of mucus clearance. This mucus layer obstructs the airways and prevents the clearance of bacteria, causing chronic infections and a severe inflammatory process. Thus, obstruction, infection, and inflammation are the three key pathologies that describe CF pulmonary symptoms. The proper design of drug delivery systems composed by matrioska formulations in that nanoparticles with mucus-penetrating properties should be incorporate into microparticles, able to be delivered by dry power inhaler could represent an important choice to treat CF. Nanoparticles should have suitable dimensions, able to diffuse through pores generated by the dense fiber mesh of mucus and proper hydrophilic and neutral surface. The material used to obtain micro matrix should be suitable to obtain other additional functions including reduction of the viscosity of the mucus. Here, nano into micro (NiMs) formulations based on mannitol or a mixture between mannitol (Man) and different helping materials, have been prepared for containing just mucus-penetrating nanocomplexes loaded with tobramycin for the treatment of Pseudomonas aeruginosa infections or containing mucus and cell-penetrating nanoparticles loaded with Ivacaftor as disease-modifying agent in CF. The specific design of tailor-made polymers for the production of nanodevices to incorporate into microparticles could represent an important strategy to obtain successful formulations of antibiotic or other drugs for pulmonary treatment of cystic fibrosis [1–3].

**References:** [1] Porsio, B.; Cusimano, B.M.G.; Schillaci, D.; Craparo, E.F.; Giammona, G.; Cavallaro, G. Nano into Micro Formulations of Tobramycin for the Treatment of Pseudomonas aeruginosa Infections in Cystic Fibrosis. *Biomacromolecules* **2017**, *18*, 3924–3935. [2] Craparo, E.F.; Porsio, B.; Schillaci, D.; Cusimano, B.M.G.; Spigolon, D.; Giammona, G.; Cavallaro, G. Polyanion-tobramycin nanocomplexes into functional microparticles for the treatment of Pseudomonas aeruginosa infections in cystic fibrosis. *Nanomedicine* **2017**, *12*, 25–42. [3] Porsio, B.; Craparo, E.F.; Nicolò, M.; Giammona, G.; Cavallaro, G. Mucus and Cell-Penetrating Nanoparticles Embedded in Nano-into-Micro Formulations for Pulmonary Delivery of Ivacaftor in Patients with Cystic Fibrosis. *ACS Appl. Mater. Interfaces* **2018**, *10*, 165–181.

## 3. Poster Presentations

### 3.1. Development of Composites Hydrogels Containing Hyaluronic Acid and Poly-Lactic-Co-Glycolic Microparticles for Cell Delivery in Regenerative Medicine

MaláZuzana^1^*ŽárskáLudmila^1^ArgentiereSimona^2^GalliAlessandra^3^PeregoCarla^3^LenardiCristina^2^1Department of Medical Biophysic, Faculty of Medicine and Dentistry, Palacky University, Hněvotínská 3, 775 15 Olomouc, Czech Republic2Department of Physics, University of Milan, Via Celoria 16, 20133 Milan, Italy3Department of Pharmacological and Biomolecular Sciences, University of Milan, Via Trentacoste 2, 20133 Milan, Italy*Correspondence: zuza.mal@seznam.cz

Hydrogels (i.e., water-swollen networks of polymeric materials) are widely used for engineering the cell microenvironment because of their high water content, biocompatibility, easy processing and tunable physicochemical properties. In this regard, it is important to develop hydrogels that mimic structures, properties and functions of native extracellular matrix (ECM) and enable cellular viability and proliferation. In this work, a composite hydrogel containing hyaluronic acid (HA) and poly-lactic-co-glycolic-acid (PLGA) microparticles (MPs) has been developed for the treatment of critical limb ischemia (CLI). The HA is a non-immunogenic, natural product that mimics the ECM, whereas the PLGA MPs have the potential to improve cell viability and proliferation due to their morpho-mechanical properties. First, MPs were prepared by emulsion polymerization using two types of PLGA polymer with two different molecular weights and lactic acid/glycolic acid ratios. After purification by centrifugation, the obtained PLGA MPs were mixed with HA gel at a concentration of 0.1%. The hydrogel composites were characterized by optical microscopy, showing different morfological features as a function of the PLGA polymer. Finally, the hydrogel composites were homogeneously mixed with fibroblasts, and their ability to maintain cell viability was assessed.

**Acknowledgments:** This work was supported by the “Regione Lombardia” under the call “LINEA R&S PER AGGREGAZIONI” with a project entitled “Cells Therapy COntrolled RElease System” (CUP: E47H16001540009).

### 3.2. Cherry Nanoparticles for the Improvement of the Protective Effect against Oxidative Stress

FabianoAngela^1^*BeconciniDenise^2^PirasAnna Maria^1^Di StefanoRossella^3^ZambitoYlenia^1^1Department of Pharmacy, University of Pisa, Via Bonanno 33, 56126 Pisa, Italy2Department of Life Sciences, University of Siena, Via P.A. Mattioli, 53100 Siena, Italy3Department of Surgerical Pathology, Medical, Molecular and Critical Area, University of Pisa, Via Savi 10, 56126 Pisa, Italy*Correspondence: angela.fabiano@for.unipi.it

The Tuscan region has shown interest in the recovery and conservation of local species in order to promote biodiversity and protect the variety of plants cultivated in the territory. Among the Tuscan agri-food products, sweet cherries from *Prunus avium* L. are popular spring-summer consumed fruits. Cherries have widely been described for their nutritional properties, as well as their content in compounds which are beneficial to human health. Cherry extract (CE) is characterized by a high content of polyphenols and, consequently, a high nutraceutical ability, which could prevent chronic diseases. Indeed epidemiological studies have suggested that fruit consumption is related to a reduction of cardiovascular disease risk factors. However, it is known that polyphenols have poor oral bioavailability due to a degradation occurring in the GI before absorption. Therefore, this study aims to evaluate the efficacy of nanoparticles (NP) as vehicles for the oral administration of antioxidants present in Tuscan CE. Previous studies reported the aptitude of NP based on chitosan derivatives for internalization by cells and improving the antioxidant activity of the entrapped polyphenols. The total phenolic content (TPC) and the antioxidant ability of CE were measured. CE-loaded NP based on two different chitosan (Ch) derivatives, i.e., quaternary ammonium-Ch (QA-Ch) and S-protected thiolated QA-Ch (S-pro-QA-Ch) conjugates, were prepared by ionotropic gelation of water-soluble precursors with hyaluronan. The two formulations were not significantly different in NP size (300–350 nm range). Also, the zeta-potential values were both positive, in agreement with the presence of quaternary ammonium ions on NP surfaces. The ex vivo studies of CE permeation across full thickness excised rat jejunum showed a permeation enhancement ratio of 1.5 with either NP type compared to the respective plain CE control. The CE entrapment efficiency in NP was always around 70%, with no differences between the two NP types. The Human Umbilical Vein Endothelial Cells (HUVECs) viability was evaluated by WST-1 assay and the reactive oxygen species (ROS) production was detected after H_2_O_2_-induced oxidative stress. CE-loaded S-pro-QA-Ch-NP showed the ability to protect HUVEC from oxidative stress (33%) and decrease ROS production (65%), probably thanks to a synergistic effect of CE and the S-protected groups present on the NP surface. The results of the present study demonstrate the ability of CE to protect the endothelial cells from oxidative stress. Moreover, CE loaded S-pro-QA-Ch-NP enhanced CE intestinal absorption and cell protection from oxidative stress.

**Acknowledgments:** Thanks to Claudio Cantini and Trees and Timber Institute-National Research Council of Italy (CNR-IVALSA) for kindly providing cherry fresh fruits.

### 3.3. Erythrocyte-Like Discoidal Nanoconstructs Carrying Tissue Plasminogen Activator for Accelerated Blood Clot

ColasuonnoMarianna^1^,^2^PalangeAnna Lisa^2^AidRachida^3^FerreiraMiguel^2^MollicaHilaria^2^,^4^PalombaRoberto^2^EmdinMichele^1^,^5^Del SetteMassimo^6^ChauvierreCédric^3^LetourneurDidier^3^DecuzziPaolo^2^*1Sant’Anna School of Advanced Studies, Piazza Martiri della Libertà, 33, 56127 Pisa, Italy2Laboratory of Nanotechnology for Precision Medicine, Fondazione Istituto Italiano di Tecnologia, Via Morego, 30, 16163 Genoa, Italy3INSERM U1148, Laboratory for Vascular Translational Science, University Paris 13, University Paris Diderot, X. Bichat Hospital, 46 rue Henri Huchard, 75018 Paris, France4Department of Informatics, Bioengineering, Robotics and System Engineering, University of Genoa, Via Opera Pia, 13, Genoa 16145, Italy5Fondazione Toscana G. monasterio, via G. Moruzzi, 1, 56124 Pisa, Italy6S.C. Neurologia, E.O. Ospedali Galliera. Mura delle Cappuccine, 14, 16128 Genova, Italy*Correspondence: paolo.decuzzi@iit.it

Tissue plasminogen activator (tPA) is the sole approved therapeutic molecule for the treatment of acute ischemic stroke. Yet, only a small percentage of patients could benefit from this life-saving treatment because of its severe side effects, including brain hemorrhages. Here, a novel thrombolytic nanoagent is realized by conjugating directly the clinical formulation of tPA to the porous structure of soft discoidal polymeric nanoconstructs (tPA-DPNs). The porous matrix of DPNs protects tPA from rapid degradation, allowing tPA-DPNs to preserve over 60% of their original activity after 3 h of exposure to serum proteins. Under dynamic conditions, tPA-DPNs dissolve clots more rapidly than free tPA, as demonstrated in a microfluidic chip where clots are spontaneously formed. mimicking in vivo conditions. Already at 60 min post treatment initiation, the clot area reduces by about 50% (57 + 8%) with tPA-DPNs, whereas a similar result (56 + 21%) is obtained after only 90 min with free tPA. In murine mesentery venules, the intravenous administration of 2.5 mg/kg of tPA-DPNs resolves almost 90% of blood clots, whereas a similar dose of free tPA successfully recanalizes only about 40% of the treated vessels. Remarkably, at about 1 tenth of the clinical dose (1.0 mg/kg), tPA-DPNs still effectively dissolve 60% of the clots, whereas tPA works properly only on 16% of the vessels. The stable conjugation and longer activity of tPA, together with the faster blood clot dissolution, would make tPA-DPNs a promising nanotool for enhancing the potency and safety of thrombolytic therapies (Figure 1).

**Acknowledgments:** This project was partially supported by the European Research Council, under the European Union’s Seventh Framework Programme (FP7/2007–2013)/ERC grant agreement no. 616695, by the Italian Association for Cancer Research (AIRC) under the individual investigator grant no. 17664, and by the European Union’s Horizon 2020 research and innovation programme under the Marie Skłodowska-Curie grant agreement No 754490. The authors acknowledge the precious support provided by the Nikon Center; the Electron Microscopy and Nanofabrication facilities at the Italian Institute of Technology.

### 3.4. Rifampicin Loaded into Inulin Based Nanomicelles Retains Its Antibacterial Effect against Mycobacteria

MandracchiaDelia^1^GrisoliPietro^2^PerteghellaSara^2^TrapaniAdriana^1^TorreMaria Luisa^2^TripodoGiuseppe^2^*1Department of Pharmacy-Drug Sciences, University of Bari “Aldo Moro”, Via Orabona 4, 70125 Bari, Italy2Department of Drug Sciences, University of Pavia, Viale Taramelli 12, 27100 Pavia, Italy*Correspondence: giuseppe.tripodo@unipv.it

Mycobacterium tuberculosis has colonized humans since the beginning of their history. It causes the tuberculosis (TB) that, even today, is hard to treat, even though effective antibiotics are available. The difficulties in treatment arise from drug resistance phenomena and from a natural protection of mycobacteria against external substances. It has been calculated that in 2012, TB caused 1.3 million of deaths worldwide [1]. One of the main causes for the incurrence of drug resistance is a low adherence of the patient to the treatment that should be carried on for approximately nine months. It has been reported that: “The repeated use of the same drugs, together with prolonged regimens that often lead to poor patient compliance, has resulted in the emergence of strains that are increasingly resistant to the available drugs” [2]. Among the drugs used for TB therapy, rifampicin is probably the most effective, but, as mentioned before, its administration, together with that of other drugs, takes months. From a formulative point of view, rifampicin shows a solubility in water of 0.14% *w*/*v*, and could therefore be considered insoluble. Thus, its formulation could present different issues for the pharmaceutical technologist, in particular, for intravenous administration. Since 2014, we have developed a drug delivery platform based on an amphiphilic derivative of inulin (INU) bioconjugated with vitamin E (VITE), called INVITE. This derivative shown great potential in loading and releasing highly hydrophobic drugs such as curcumin or celecoxib [3,4]. This derivative also showed long-circulating features after intravenous administration [4,5]. Based on these studies, we had the idea to load rifampicin into the INVITE nanomicelles and test these drug delivery systems for their activity against Mycobacterium smegmatis. which could be considered a good model, i.e., one that is less hazardous to manipulate with respect to Mycobacterium tuberculosis, to assess the antibacterial activity of our systems. As previously mentioned, INU was chemically-derived with VITE, and the obtained INVITE bioconjugate was allowed to form the nanomicelles, and loaded with the selected drug by the dialysis method. The drug-loaded micelles were lyophilized, and the gained powder was easily redispersed, allowing us to obtain a homogeneous dispersion in water or phosphate buffer of the insoluble drug. The calculated drug loading was 6% *w*/*w*. The antimicrobial activity of the rifampicin loaded micelles was determined with the macrodilution broth method, according to Clinical and Laboratory Standards Institute. The results returned an effective antibacterial activity, comparable to that of free rifampicin. This indicates that the drug does not show any loss in activity when included in INVITE nanomicelles, thereby showing that the developed drug delivery system is an important tool in a prospective use of it in the therapy of TB.

**References:** [1] Glaziou, P.; Sismanidis, C.; Floyd, K.; Raviglione, M. Global Epidemiology of Tuberculosis. *Cold Spring Harb. Perspect. Med.* **2015**, *5*, a017798. [2] Smith, T.; Wolff, K.A.; Nguyen, L. Molecular biology of drug resistance in Mycobacterium tuberculosis. *Curr. Top. Microbiol. Immunol.* **2013**, *374*, 53–80. [3] Mandracchia, D.; Trapani, A.; Perteghella, S.; Sorrenti, M.; Catenacci, L.; Torre, M.L.; Trapani, G.; Tripodo, G. pH-sensitive inulin-based nanomicelles for intestinal site-specific and controlled release of celecoxib. *Carbohydr. Polym.* **2018**, *181*, 570–578. [4] Tripodo, G.; Pasut, G.; Trapani, A.; Mero, A.; Lasorsa, F.M.; Chlapanidas, T.; Trapani, G.; Mandracchia, D. Inulin-d-α-tocopherol succinate (INVITE) nanomicelles as a platform for effective intravenous administration of curcumin. *Biomacromolecules* **2015**, *16*, 550–557. [5] Mandracchia, D.; Rosato, A.; Trapani, A.; Chlapanidas, T.; Montagner, I.M.; Perteghella, S.; di Franco, C.; Torre, M.L.; Trapani, G.; Tripodo, G. Design, synthesis and evaluation of biotin decorated inulin-based polymeric micelles as long-circulating nanocarriers for targeted drug delivery. *Nanomedicine* **2017**, *13*, 1245–1254.

### 3.5. Compounding of (Trans)Dermal Patches by Hot-Melt Ram Extrusion 3D Printing

MusazziUmberto M.*GennariChiara G.M.MinghettiPaolaCilurzoFrancescoDepartment of Pharmaceutical Sciences, University of Milan, Via. G. Colombo, 71, 20122 Milano, Italy*Correspondence: umberto.musazzi@unimi.it

(Trans)dermal patches (TP) are well-known pharmaceutical preparations designed to provide prolonged drug delivery through the skin to achieve a local, regional or systemic effect. TPs are often preferred to other topically-applied dosage forms since they make it possible to predetermine the drug absorption kinetic and to define the treated area. Thus, TPs can reduce the side effects on healthy skin and due to an undesired systemic drug absorption when localized cutaneous diseases or injuries have to be treated. TPs are produced by a solvent casting technique, but they cannot be easily compounded since, after solvent evaporation, significant modifications of the adhesive matrix, and therefore, of the drug release and adhesive properties, can occur over an unknown period of time, ranging from some days to weeks. These alterations cannot be monitored in a pharmacy setting.

This work demonstrated the feasibility of the extemporaneous preparation of (trans)dermal patches by hot-melt ram extrusion 3D printing [1]. This technology makes it possible to easily define both the patch geometry and the dose according to patient needs. The TP preparation consists of three simple technological operations: (i) the drug, the film-forming material (Eudragit (Eu) RL, RS or blends thereof) and the plasticizer (triacetin, TRI, or try-butyl citrate, TBC), which confers the adhesive properties [2], are mixed in a mortar; (ii) the mixture is fed in to the chamber of the ram-extruder and heated to 90 °C; (iii) the melt mixture is printed with the desired geometry (thickness: 50 μm) on the backing layer and coupled with the release liner. The adhesive properties of printed patches were investigated by shear and 180°-peel adhesion tests. The results showed that patches with suitable adhesive properties can be printed using 40% *w*/*w* of TRI or 50% *w*/*w* of TBC. The TRI-containing patches showed higher shear adhesion values than TBC ones (*p* < 0.05). Since high values of shear adhesion are essential for the patch permanence onto the skin, TRI (40% *w*/*w*) was selected to print drug-loaded patches, using 2.34% *w*/*w* of ketoprofen (KP) and 3% of nicotine (NT) as model compounds. Neither drug affected the patch adhesive properties, even if a reduction of shear adhesion up to 8-folds was observed based on the drug type and the EuRL/EuRS ratio. Finally, the in vitro release studies showed that the EuRL/EuRS ratio impacted significantly on the release rate of both the tested drugs. According to the well-known characteristics of the two copolymers, the higher the concentration of EuRL in the matrix, the higher the release rate of both KP and NT.

**References:** [1] Musazzi, U.M.; Selmin, F.; Ortenzi, M.A.; Mohammed, G.K.; Franzé, S.; Minghetti, P.; Cilurzo, F. Personalized orodispersible films by hot melt ram extrusion 3d printing. *Int. J. Pharm.* **2018**, *551*, 52–59. [2] Quaroni, G.M.G.; Gennari, C.G.M.; Cilurzo, F.; Guylaine, D.; Creton, C.; Minghetti, P. Tuning the rheological properties of an ammonium methacrylate copolymer for the design of adhesives suitable for transdermal patches. *Eur. J. Pharm. Sci.* **2018**, *111*, 238–246.

### 3.6. Preparation and Characterization of Water Soluble Carvacrol Prodrug-Clay Hybrids

PreziusoFrancesca^1^*EusepiPiera^1^Di StefanoAntonio^1^CacciatoreIvana^1^CiullaMichele^1^MarinelliLisa^1^GenoveseSalvatore^1^EpifanoFrancesco^1^AguzziCarola^2^ViserasCesar^2^,^3^1Department of Pharmacy, University “G. d’Annunzio” of Chieti—Pescara, 66100 Chieti, Italy2Department of Pharmacy and Pharmaceutical Technology, School of Pharmacy, University of Granada, 18071 Granada, Spain3Instituto Andaluz de Ciencias de la Tierra, CSIC-University of Granada, 18071 Granada, Spain*Correspondence: francesca.preziuso@unich.it

Pharmaceutical-grade clay minerals are widely used to encapsulate drugs, overcoming undesirable physicochemical properties or modifying the release kinetic of active compounds [1,2]. Among clay minerals, tubular Halloysite (HAL), stratified Montmorillonite (VHS), fibrous Sepiolite (SPT) and Palygorskite (PC) are largely used in the pharmaceutical field [3]. In our study, the prodrug approach was employed in the rational drug design of novel derivatives to improve carvacrol (CAR) solubility while still retaining the antibacterial activity. Preliminary results showed that WSCP1, WSCP2 and WSCP3 (CAR amino-acid esters) were the most active compounds against Gram positive bacteria, but were endowed with low plasma stability. The aim of this work was the adsorption of these three CAR derivatives on different clay minerals as a prior step to developing an adequate delivery system. Additionally, a complete characterization of the novel hybrids was conducted to elucidate the nature and degree of prodrug interactions with minerals clays. High performance liquid chromatography results and thermal analysis revealed that among the tested clay minerals, SPT and HAL retained lower amounts of the drug. Conversely, VHS followed by PC possessed the higher loading capacities, ranging from 20–25% for WSCP1- and WSCP3-VHS hybrids, 18–20% for WSCP1- and WSCP3-PC, and approximately 45% and 27% for WSCP2 loaded in VHS and PC. Considering the higher drug loading values, VHS hybrids were selected for further studies. X-Ray powder diffraction analysis confirmed the effective inclusion of prodrugs into the VHS interlayer by shifting of VHS basal spacing value. Fourier transform infrared spectra corroborated the interaction between the organic and inorganic components through the revelation of new bonds in the hybrid samples. Release studies carried out at pH 1.2 and 6.8 buffers revealed that the desorbed drug increased quickly in the first hour of the experiments until reaching a plateau phase in which the percentage of WSCPs in the buffer media remained constant for 8 h. After adsorption into VHS, a higher stability of WSCPs was achieved, especially at pH 6.8 phosphate buffer, thanks to the delayed release over time. In conclusion, adsorption of CAR derivatives onto pharmaceutical-grade clay minerals was successfully achieved, especially on VHS. In addition, WSCP stability in physiological conditions was improved due to the intercalation of WSCP1-3 into VHS interlayers, which protected the drugs from hydrolysis in the gastrointestinal simulated fluids.

**References:** [1] Aguzzi, C.; Cerezo, P.; Viseras, C.; Caramella, C. Use of clays as drug delivery systems: possibilities and limitations. *Appl. Clay Sci.* **2007**, *36*, 22–36. [2] Sandri, G.; Bonferoni, M.C.; Rossi, S.; Ferrari, F.; Aguzzi, C.; Viseras, C.; Caramella, C. Chapter 19: Clay minerals for tissue regeneration, repair, and engineering. In *Wound Healing Biomaterials*; Elsevier: Amsterdam, The Netherlands, 2016. [3] López-Galindo, A.; Viseras, C.; Aguzzi, C.; Cerezo, P. Pharmaceutical and cosmetic uses of fibrous clays. In *Developments in Clay Science*; Elsevier: Amsterdam, The Netherlands, 2011; pp. 299–324.

### 3.7. Hyaluronan-Based Nanogels as Trojan Horse: Chasing Intracellular Pathogens

MontanariElita^1^ZorattoNicole^1^Di MeoChiara^1^CovielloTommasina^1^ManciniPatrizia^2^MoscaLuciana^3^MatricardiPietro^1^*1Department of Drug Chemistry and Technologies, Sapienza University, 00185 Rome, Italy2Department of Experimental Medicine, Sapienza University, 00185 Rome, Italy3Department of Biochemical Sciences, Sapienza University, 00185 Rome, Italy*Correspondence: pietro.matricardi@uniroma1.it

*Staphylococcus aureus* is one of the most significant human pathogens that is frequently isolated in a wide range of superficial and systemic infections. The ability of *S. aureus* to invade and survive within host cells such as keratino-cytes and host immune cells has been increasingly recognized as a potential factor in persistent infections and treatment failures. *Staphylococcus aureus* is a major human pathogen that causes a wide range of superficial and systemic infections (e.g., nares, respiratory tract and skin infections). Because of its ability to invade and survive within host cells, such as keratinocytes and immune cells, S. aureus is nowadays considered a facultative intracellular pathogen [1]. This adaptive mechanism is a potential factor in persistent infections and treatment failure, due to the lack of access of the antibiotics to the intracellular site of infection. The aim of this work was to develop antimicrobial-loaded hyaluronan cholesterol nanohydrogels (HA-CH NHs), for enhancing the intracellular uptake of antibiotics into human keratinocytes, in order to target intracellular *S. aureus* [2]. In this context, the application of HA-CH nanocarriers may be a successful strategy thanks to the ability of HA to enter cells through CD44 receptor (highly expressed in keratinocytes), which is also employed by a number of pathogens [3]. Moreover, these NHs can be engineered to reach desired cellular compartments, facilitating the pathogen targeting. For this purpose, sterile and self-assembled gentamicin (GM)- or levofloxacin (LVF)-loaded NHs were obtained with a fast, one-step autoclaving process (121 °C, 20′), according to a method that was previously reported [4]. GM and LVF-loaded NHs showed mean hydrodynamic diameter of ~250 and 300 nm, respectively. Neither formulation affected the viability of human keratinocytes (HaCaT) at all the tested concentrations over 48 h, and exhibited the same minimum inhibitory concentration (MIC) and minimum bactericidal concentration (MBC) values as free antibiotics against extracellular *S. aureus*. However, the intracellular antibacterial activity of LVF was deeply enhanced by NHs. LVF is predominantly a cytosolic drug; however, thanks to NH’s ability to colocalize with lysosomes of HaCaT cells, LVF-NHs may be able to change the intracellular fate of LVF from cytosol to lysosome, thereby targeting to intracellular *S. aureus* which is known to accumulate predominantly in lysosomes. In contrast, GM showed a significant intracellular activity without the use of NHs due to its ability to accumulate in lysosomes. This work opens new perspectives in the development of more effective formulations for the treatment of persistent *S. aureus* skin infections.

**Acknowledgments:** The research was partially supported by “Finanziamenti di Ateneo per la Ricerca Scientifica—RP116154C2EF9AC8” and “Progetto di Ricerca RM11715C1743EE89”, Sapienza University of Rome.

**References:** [1] Rollin, G.; Tan, X.; Tros, F.; Dupuis, M.; Nassif, X.; Charbit, A.; Coureuil, M. Intracellular survival of Staphylococcus aureus in endothelial cells: A matter of growth or persistence. *Front. Microbiol.* **2017**, *8*, 1354. [2] Montanari, E.; Oates, A.; di Meo, C.; Meade, J.; Cerrone, R.; Francioso, A.; Devine, D.; Coviello, T.; Mancini, P.; Mosca, L.; et al. Hyaluronan-Based Nanohydrogels for Targeting Intracellular *S. Aureus* in Human Keratinocytes. *Adv. Healthc. Mater.* **2018**, *7*, 1701483. [3] Leemans, J.C.; Florquin, S.; Heikens, M.; Pals, S.T.; van der Neut, R.; van der Poll, T. CD44 is a macrophage binding site for Mycobacterium tuberculosis that mediates macrophage recruitment and protective immunity against tuberculosis. *J. Clin. Investig.* **2003**, *111*, 681–689. [4] Montanari, E.; de Rugeriis, M.C.; di Meo, C.; Censi, R.; Coviello, T.; Alhaique, F.; Matricardi, P. One-step formation and sterilization of gellan and hyaluronan nanohydrogels using autoclave. *J. Mater. Sci. Mater. Med.* **2015**, *26*, 32.

### 3.8. Enzyme Loaded PLGA Nanoparticles; A Delicate Balance of Technological Variables

PederzoliFrancesca^1^,^2^RuoziBarbara^2^OddoneNatalia^2^TomaninRosella^1^RigonLaura^1^D’AvanzoFrancesca^1^VandelliMaria Angela^2^DuskeyJason^2^ForniFlavio^2^TosiGiovanni^2^*1Istututo di Ricerca Pediatrico, Città della Speranza, Corso Stati Uniti 4, 35127 Padova, Italy2Department of Life Science, University of Modena and Reggio Emilia, via Campi 103, 41125 Modena, Italy*Correspondence: gtosi@unimore.it

Polymeric nanoparticles (NPs) have been shown to be promising carriers for enzyme delivery due to their potential in both protecting drugs and modulating the release at the target site. However, several technological aspects should be considered critical to obtaining high loading of active enzyme, as the NP preparation process often impairs enzyme stability. In this context, this research focused on technological variables in NP formulation in order to stabilize enzyme active, thus increasing efficacy. Poly-lactic-co-glycolic acid (PLGA) NPs were obtained by water-in oil-in water (W1/O/W2) double emulsion preparation protocol. -glucosidase was used as model enzyme. Several pre-formulative studies were carried out (i) to drastically reduce mechanical and energetic stress factors caused by preparative procedures (e.g., sonication); (ii) to optimize the volume of liquid phases; and (iii) to choose type of surfactant and concentration. In order to better preserve enzyme integrity, the presence/absence of stabilizing excipients (BSA, trehalose and PEG) were also experimented in enzyme loaded NPs. Spectroscopical (PCS) and microscopical (AFM, STEM) techniques were used for chemico-physical characterization of samples. For each preparation, enzyme loading was evaluated after extraction and analytical quantification by HPLC, and enzyme stability was monitored by enzymatic activity assay. Preformulative studies suggested that w/o/w emulsion is a very critical step; in particular, these studies defined the limits in terms of power and time of sonication (hypothetical dangerous steps to lead enzyme destabilization), the W1/O/W2 volume ratio and the % of surfactant in W2 in order to keep monomodal and monodisperse NPs (S:60 Watt/30″; W1/O/W2 1/2/4; PVA 1%). On these bases, the different formulation processes designed to allow enzyme encapsulation were planned, also taking into account the use of several stabilized agents to preserve the enzyme activity. In particular, the addition of BSA (in the inner water phase) exhibited both an increased rate of encapsulation as well as a stabilizing effect.

In vitro experiments are ongoing to evaluate the release kinetic of enzyme and the stability of these formulations over time. The high loading capacity obtained by using enzyme model drugs with preserved enzymatic activity was an important starting point to formulate NPs with optimized conditions for therapeutic enzyme loading.

**Acknowledgments:** The authors refer no conflict of interest. The study was performed with funding support of Fondazione Cassa di risparmio di Padova e Rovigo.

### 3.9. Combination of Immune Checkpoint Blockade with DNA Cancer Vaccine Induces Potent Antitumor Immunity against P815 Mastocytoma

LopesAlessandra^1^VanvarenbergKevin^1^KosŠpela^2^PréatVéronique^1^*†VandermeulenGaëlle^1^†1Louvain Drug Research Institute, Advanced Drug Delivery and Biomaterials, Université Catholique de Louvain, B-1200 Brussels, Belgium2Department of Experimental Oncology, Institute of Oncology Ljubljana, Zaloska 2, SI-1000 Ljubljana, Slovenia*Correspondence: veronique.preat@uclouvain.be†These authors contributed equally to this work.

DNA vaccination against cancer has become a promising strategy for inducing a specific and long-lasting antitumor immunity. However, DNA vaccines fail to generate potent immune responses when used as a single therapy. To enhance their activity in the tumor, a DNA vaccine against murine P815 mastocytoma was combined with antibodies directed against the immune checkpoints CTLA4 and PD1, which are involved in T-cell activity. The combination of these two strategies delayed tumor growth and enhanced specific antitumor immune cell infiltration in comparison to the corresponding single therapies. The combination also promoted IFNg, IL12 and granzyme B production in the tumor microenvironment and decreased the formation of liver metastasis in a very early phase of tumor development, enabling 90% survival. These results underline the complementarity of DNA vaccination and immune checkpoint blockers in inducing a potent immune response by exploiting the generation of antigen-specific T cells by the vaccine and the ability of immune checkpoint blockers to enhance T-cell activity and infiltration in the tumor. These findings suggest how and why a rational combination therapy can overcome the limits of DNA vaccination, but could also allow responses to immune checkpoint blockers in a larger proportion of subjects.

**Acknowledgments:** G.V. is supported by a FIRST spin-off grant 1610437 from the Walloon Region.

### 3.10. Protein Functionalized Solid Lipid Nanoparticles to Improve Brain Biodistribution of Methotrexate

MuntoniElisabetta^1^BattagliaLuigi^1^*MariniElisabetta^1^GiorgisMarta^1^LazzaratoLoretta^1^SalaroglioIris Chiara^2^RigantiChiara^2^MartinaKatia^1^1Dipartimento di Scienza e Tecnologia del Farmaco, Università degli Studi di Torino, via Pietro Giuria 9, 10124 Torino, Italy2Dipartimento di Oncologia, Università degli Studi di Torino, Regione Gonzole 10, 10043 Orbassano, Italy*Correspondence: luigi.battaglia@unito.it

Glioblastoma Multiforme (GB) is the most common and invasive primary central nervous system (SNC) tumors, with a very poor prognosis. The brain-blood barrier (BBB) is the main obstacle to GB pharmacological treatment. Nanoparticles emerged as versatile vectors that can overcome the BBB, particularly through the active targeting strategies. In this experimental work, solid lipid nanoparticles (SLN), prepared by fatty acid coacervation [1], were loaded with an active lipophilic ester of methotrexate (MTX), didodecylmethotrexate (ddMTX) [2], and functionalized with transferrin and insulin, two proteins whose receptors are abundantly expressed on the BBB. Functionalization was achieved by grafting on the SLN surface a maleimidic moiety and exploiting its reactivity towards thiolated proteins. The derivatization was confirmed by SDS-PAGE followed by Blue Coomassie staining. BBB overcoming of ddMTX loaded SLN was tested in vitro on cellular models and in vivo on Wistar rats through bioditribution studies. Drug metabolism, in particular the presence of 7-hydroxymethotrexate (7OH-MTX)—the only active metabolite of MTX—was investigated in the animal model by mass spectroscopy. The data obtained, although preliminary, are interesting: ddMTX-SLN functionalizated with PEGilated linkers improved overcoming BBB in cell models, as well as in rats. The drug was extensively metabolized in vivo, but 7OH-MTX was not the major metabolite in the model under study.

Further studies on in vivo glioma models will be performed in order to evaluate the potential application of this approach to GB therapy.

**Acknowledgments:** Ricerca Locale 2017–2018, Compagnia di San Paolo 2011.

**References:** [1] Battaglia, L.; Gallarate, M.; Cavalli, R.; Trotta, M. Solid lipid nanoparticles produced through a coacervation method. *J. Microencapsul.* **2010**, *27*, 78–85. [2] Rosowsky, A.; Forsch, R.A.; Yu, C.S.; Lazarus, H.; Beardsley, G.P. Methotrexate analogues. 21. Divergent influence of alkyl chain on the dihydrofolate reductase affinity and cytotoxicity of methotrexate monoesters. *J. Med. Chem.* **1984**, *27*, 605–609.

### 3.11. β-Cyclodextrin Nanosponges for Enabling ICOS Antitumor Effect

ArgenzianoMonica^1^*DianzaniChiara^1^FerraraBenedetta^1^ClementeNausicaa^2^CalderaFabrizio^3^TrottaFrancesco^3^DianzaniUmberto^2^CavalliRoberta^1^1Department of Drug Science and Technology, University of Turin, Via P. Giuria 9, 10125 Torino, Italy2Department of Life Sciences, Università del Piemonte Orientale, via Solaroli 17, 28100 Novara, Italy3Department of Chemistry, University of Turin, Via P. Giuria 7, 10125 Torino, Italy*Correspondence: monica.argenziano@unito.it

ICOS is a T cell co-stimulatory molecule involved in T cell function. It binds B7h expressed by several cell types including tumor cells. B7h triggering by ICOS modulates cytokine secretion in dendritic cells, inhibits adhesiveness and migration of dendritic, endothelial and tumor cells [1]. B7h:ICOS interaction may modulate the spread of cancer metastases, suggesting the novel use of ICOS-Fc as an immunomodulatory drug. In melanoma, treatment with anti-CTLA-4 Ab induces an increase of ICOS+ effector T cells, indicating that the ICOS/B7h pathway is required for antitumor responses [1]. However, in vivo administration of free ICOS-Fc solution induces only anti-metastasis effects, without inhibiting the local growth of B16 cells injected subcutaneously in mice. In this work, ICOS-Fc was incorporated in β-cyclodextrin based nanosponges (NS), with the aim of increasing its delivery to the tumor, enabling an anticancer effect. NS are innovative, polymer-based delivery systems consisting of cross-linked cyclodextrins nanostructured within a three-dimensional network [2]. They were synthetized by reacting β-cyclodextrin and pyromellitic dianhydride as cross-linking agent. To obtain an aqueous NS nanosuspension suitable for drug delivery, a top-down method was tuned. The NS sample underwent high pressure homogenization (HPH) to reduce the size of the NSs and obtain an almost-homogenous nanoparticle distribution. ICOS-Fc was incorporated in NSs by incubation at room temperature without the addition of any solvent. NS formulations, either blank or loaded, were characterized by size, surface charge, morphology analyses and in vitro release studies. In vivo experiments were performed using the B16 melanoma model of transplantable tumors. C57BL/6 mice were injected subcutaneously with 105 B16-F10 cells and treated with an i.v. injection of either the mouse ICOS-Fc, ICOS-Fc loaded in NS or the empty NS as control. An aqueous nanosuspension of NS with an average diameter of about 300 nm and low polydispersity index was obtained. ICOS-Fc was loaded into the NS with a good encapsulation efficiency. A slow and prolonged in vitro release kinetics of ICOS-Fc from the nanoformulation was observed. The loaded NS nanoformulation was stable for more than 6 months, protecting ICOS-Fc from degradation. In vivo experiments showed that i.v. injection of ICOS-Fc loaded in NS remarkably inhibited either the metastasis formation or the growth of established subcutaneous B16 tumors. Interestingly, the delivery of ICOS-Fc with NS is crucial for its therapeutic effectiveness. This result showed for the first time that the combination of ICOS with a nanocarrier can enhance its antitumor response.

**References:** [1] Gigliotti, C.L.; Boggio, E.; Clemente, N.; Shivakumar, Y.; Toth, E.; Sblattero, D.; D’Amelio, P.; Isaia, G.C.; Dianzani, C.; Yagi, J.; et al. ICOS-ligand triggering impairs osteoclast differentiation and function in vitro and in vivo. *J. Immunol.* **2016**, *197*, 3905–3916; [2] Trotta, F.; Dianzani, C.; Caldera, F.; Mognetti, B.; Cavalli, R. The application of nanosponges to cancer drug delivery. *Expert Opin. Drug Deliv.* **2014**, *11*, 931–941.

### 3.12. Exploring the Use of Spray Congealing to Produce Solid Dispersions with Enhanced Indomethacin Bioavailability

BertoniSerena^1^*DalpiazAlessandro^2^FerraroLuca^3^AlbertiniBeatrice^1^PasseriniNadia^1^1Department of Pharmacy and BioTechnology, PharmTech Lab, University of Bologna, Via S. Donato 19/2, 40127 Bologna, Italy2Department of Chemical and Pharmaceutical Sciences, University of Ferrara, Via Fossato di Mortara 19, 44121 Ferrara, Italy3Department of Life Sciences and Biotechnology, University of Ferrara, via L. Borsari 46, 44121 Ferrara, Italy*Correspondence: serena.bertoni4@unibo.it

The potential risk of drug re-crystallization is a serious concern for the stability of amorphous systems, and represents, despite the great bioavailability, one of the main factors that limits their clinical application. Moreover, besides reproducible in vitro and in vivo performance, easily scaled-up manufacturing technology, non-toxic and low-cost formulation and prolonged stability are also important prerequisites for a successful formulation. This research proposes an original oral delivery system for the bioavailability enhancement of indomethacin (IND), with the aim of overcoming the common limitations of the amorphous solid dispersion in view of an industrial application. To this end, IND-loaded microparticles (MPs) were prepared by spray congealing using oral-approved excipients (Gelucire 50/13 and the recently marketed Gelucire 48/16 at different weight ratio). MPs were characterized regarding particle size, morphology, drug content, drug solubility and dissolution rate. A solid-state characterization (FT-IR, DSC, HSM and PXRD) was also carried out. In in vivo studies, male Sprague−Dawley rats received a femoral intravenous infusion of IND or oral administrations of MPs and free IND; the plasma of rats was analyzed by HPLC over time in order to evaluate the absolute bioavailability of oral doses. Spray congealed MPs loaded with 10% of IND showed encapsulation efficiency values close to 100%. IND solubility and dissolution rates markedly increased by the incorporation into spray-congealed MPs. Specifically, the formulation containing Gelucire 50/13 and Gelucire 48/16 at 3:7 weight ratio showed the best in vitro performance, leading to a significant increase of both drug solubility and dissolution rate compared both with the pure drug and the correspondent physical mixture. FT-IR, DSC and PXRD demonstrated the transformation of the drug into the amorphous phase in spray-congealed MPs. This solid-phase transition contributed, together with the wetting effect of the hydrophilic excipients, to the enhancement of solubility and dissolution behavior. Notably, IND amorphous state and dissolution profiles of the MPs were unchanged after 18 months of storage, confirming the physical stability of the systems. In vivo studies indicated a value of about 20% for the oral bioavailability of solid pure IND; the loaded spray congealed MPs showing the best in vitro performance made it possible to obtain an increase of IND bioavailability of up to about 50%. Further studies are in progress in order to evaluate in vivo the behavior of the physical mixture.

The MPs produced by spray congealing improved IND bioavailability, and thus, the developed system could be applicable to the optimization of its clinical use by decreasing the required daily dose. This underlines an area for further investigation regarding the application of Gelucire 48/16-based spray congealed MPs to different BCS class II drugs, as well as insights into the mechanisms of the bioavailability enhancement.

### 3.13. Albumin-Based Nanoparticles for Improving Intracellular Delivery of Doxorubicin in Resistant Cancer Cell Lines

BessoneFedericaFerraraBenedettaDianzaniChiaraCavalliRoberta*Department of Drug Science and Technology, University of Turin, Via P. Giuria 9, 10125 Turin, Italy*Correspondence: roberta.cavalli@unito.it

Resistance to chemotherapy is a major problem that limits the effectiveness of cancer treatment. The anthracycline doxorubicin (DOXO) is an antineoplastic agent used in the treatment of a wide range of cancers, such as multiple myeloma, lung, ovarian, gastric, thyroid, breast, sarcoma, and pediatric cancer. Used in the majority of chemotherapies, DOXO is renowned for its extensive side-effect profile, in particular the dose-dependent cardiotoxicity, the lack of selectivity for tumor cells and the induced cell resistance. Nanotechnology-based drug delivery systems are a promising alternative to overcome different limitations in cancer therapy. The nanoparticles increase local drug concentration and control its release in a sustained and controlled manner. Albumin is suitable as a material for preparing nanoparticles [1,2]. In this work, albumin-based nanoparticles loading DOXO were formulated with the aim of providing a controlled release of the drug to reduce the toxicity and to overcome DOXO resistance. Glycol chitosan-coated (GC DOXO-NP) and un-coated (DOXO-NP) nanoparticles were obtained with a tuned coacervation method. The formulations were characterized by measuring the size, the polydispersity index and the zeta potential by laser light scattering. Transmission electron microscopy (TEM) was used to study nanoparticle morphology. The formulation stability was confirmed in the short and long term. DOXO was encapsulated in a great extent and was released from the nanoparticles with prolonged in vitro release kinetics. Biological assays were performed on A2780 res and EMT6, respectively human ovarian carcinoma and mouse mammary cell lines resistant for DOXO. Cell viability assays (MTT test) were carried out and the nanoparticles showed higher cytotoxicity than the free drug after 24 and 48 h of incubation. Moreover, the cell reproductive death after treatment with the nanoparticles at different times was determined by clonogenic assay. At low concentrations, both DOXO-NP and GC DOXO-NP inhibited the cell colony formation to a greater extent than DOXO solution. In addition, the cell uptake of the different nanoparticle formulations was evaluated by confocal microscopy. Taken together, these results showed that albumin-based DOXO-loaded nanoparticles might represent a novel platform to overcome the mechanism of drug resistance in cancer cell lines and improve the efficacy of the chemotherapy.

**References:** [1] Bhushan, B.; Khanadeev, V.; Khlebtsov, B.; Khlebtsov, N.; Gopinath, P. Impact of albumin based approaches in nanomedicine: Imaging, targeting and drug delivery. *Adv. Colloid Interface Sci.* **2017**, *246*, 13–39. [2] Zhitnyak, I.; Bychkov, I.; Sukhorukova, I.V.; Kovalskii, A.M.; Firestein, K.; Golberg, D.; Gloushankova, N.; Shtansky, D.V. Effect of BN Nanoparticles Loaded with Doxorubicin on Tumor Cells with Multiple Drug Resistance. *ACS Appl. Mater. Interfaces* **2017**, *9*, 32498–32508.

### 3.14. NLC for Mediterranean Essential Oils delivery

CarboneClaudia^1^,^2^*MusumeciTeresa^1^CaddeoCarla^3^PignatelloRosario^1^PuglisiGiovanni^1^SoutoEliana B.^2^,^4^1Laboratory of Drug Delivery Technology, Department of Drug Sciences, University of Catania, 95131 Catania, Italy2Department of Pharmaceutical Technology, Faculty of Pharmacy, University of Coimbra (FFUC), 3000-548 Coimbra, Portugal3Department of Life and Environmental Sciences, Sezione di Scienze del Farmaco, University of Cagliari, 09124 Cagliari, Italy4REQUIMTE/LAQV, Group of Pharmaceutical Technology, Faculty of Pharmacy, University of Coimbra, 3000-548 Coimbra, Portugal*Correspondence: ccarbone@unict.it

Mediterranean *Essential oils* (EOs) such as *Origanum* spp., *Thymus* spp. and *Rosmarinus* spp., commonly used in the food industry, are recognized as valuable active pharmaceutical ingredients due to their antibacterial, antifungal, antiviral, antioxidant, anticancer, immune-modulatory, analgesic and anti-inflammatory activities. Nevertheless, EO use in pharmaceutics is limited by the low water solubility and stability (i.e., volatility, oxidation). Based on that, we aimed to exploit the nanoencapsulation through nanostructured lipid carriers (NLC) as a potential valid strategy. Among Mediterranean EOs, we selected *Rosmarinus officinalis* L., Lavandula x intermedia “Sumian”, *Origanum vulgare* subsp. hirtum and *Thymus capitatus* as intrinsic matrix components and active ingredients of NLC prepared by phase inversion temperature (PIT) and high-pressure homogenization (HPH) methods, using Softisan as solid lipid and two different surfactants mixtures. NLC systems were characterized in terms of mean particles size and polydispersity (Zetasizer, Malvern Instrument, Worcestershire, UK), chemical structures by FT-IR spectrophotometer (Perkin Elmer Spectrum RX I, Waltham, MA, USA), stability (Turbiscan^®^ AGS, Formulaction, France), in vitro cell viability and anti-inflammatory activities on Raw 264.7 cells (macrophage cell line) and in vitro antioxidant activity by DPPH assay. The optimized NLC obtained with the mixture Kolliphor/Labrafil using Rosmarinus, Lavandula and Origanum EOs, showed a single peak in the distribution (PDI < 0.15) of small sized nanoparticles (Zave < 200 nm). Turbiscan technology was exploited to evaluate the effect of the production method and surfactant mixture composition on the longterm stability of EOs-loaded NLC. In vitro biological studies showed that Lavandula and Rosmarinus NLC were the most biocompatible formulations up to a concentration of 0.1% (*v*/*v*). A dose-dependent anti-inflammatory activity was observed in the order Lavandula > Rosmarinus ≥ Origanum, both as free oils or loaded into NLC, the latter being the only component with a significant concentration-dependent free-radical scavenging activity. Taken together, these results allowed us to infer that Mediterranean EOs, thanks to their relevant anti-inflammatory (or antioxidant) activity, can be proposed as both active ingredients and oily matrix components of NLC, prepared by the lab-scale PIT method or the scalable HPH method, without significant differences in their physico-chemical properties, thus enhancing the biocompatibility and reducing the cytotoxicity of the bioactive EOs.

**Acknowledgments:** This work was supported by Research Funding for University of Catania, under Project Piano per la Ricerca 2016–2018—Linea Di Intervento 2 “Dotazione Ordinaria” cod. 57722172106.

### 3.15. Preliminary Results on the Preparation of Spanish Broom, Flax and Hemp Wound Dressings Based on 18-β-Glycyrrhetinic Acid-Cyclodextrin Inclusion Complexes

CerchiaraTeresa^1^*AbruzzoAngela^1^DallaFrancesco^1^DalenaFrancesco^2^De LucaGiuseppina^2^GiordaniBarbara^1^BigucciFederica^1^LuppiBarbara^1^1Pharmacy and Biotechnology Dpt., Bologna University, Via San Donato 19/2, 40127 Bologna, Italy2Chemistry and Chemical technologies Dpt., Calabria University, Via P. Bucci, 87036 Arcavacata di Rende, Cosenza, Italy*Correspondence: teresa.cerchiara2@unibo.it

Each year, acute as well as chronic skin wounds affect millions of people around the world, influencing their quality of life [1]. Moreover, the management of wounds is very costly to health services, and the choice of dressing varies depending on the wound’s characteristics. Most commonly, cotton gauze is used thanks to its intrinsic properties, i.e., high hydrophilicity and absorbing ability. Cotton farming involves environmental risks due to the intensive use of pesticides and the large amounts of water required for cotton cultivation, causing soil desalinization and degradation of soil fertility. Taking into account these disadvantages, we investigated the potential use of Spanish Broom (SB), flax (F) and hemp (H) dressings for wound care as a good alternative to cotton gauzes. SB, F and H fibers, as well as cotton fibers, are composed of cellulose and have hydrophilic character. In order to yield healing activity, natural products like propolis, Aloe vera, hyaluronic acid, vitamin E and glycyrrhetinic acid (GA) can be used to improve wound healing process. It is well known that GA can be utilized to treat various diseases including wounds and ulcers [2]. It is a lipophilic drug with a very low solubility in water. In this work, in order to improve its water solubility, we reported the preparation and characterization of the inclusion complexes formed by GA and 2-HP-γ-CD or 2-HP-β-CD. The inclusion complexes were investigated in solution by phase solubility diagrams and 1H-NMR spectrometry and in solid state by FT-IR spectroscopy. Additionally, three different wound dressings based on SB, F and H loaded with GA-cyclodextrin inclusion complex with or without hyaluronic acid gel were prepared. These formulations were characterized in terms of morphology by Scanning Electron Microscopy (SEM) and in vitro release studies. The results showed that both cyclodextrins increased the water solubility of GA particularly 2-HP-γ-CD. SEM micrographs showed that SB, F and H wound dressings were homogeneously covered with hyaluronic gel containing GA-cyclodextrin inclusion complex. Finally, in vitro release studies showed that GA was vastly released from SB, F and H wounds dressings, while a slower release was obtained from SB, F and H wound dressings in the presence of hyaluronic acid gel. In conclusion, SB, F and H wound dressings loaded with GA-cyclodextrin inclusion complex can be used as new delivery systems for the treatment of skin wounds.

**Acknowledgments:** This study was supported by Fondazione Cassa di Risparmio di Imola.

**References:** [1] Qureshi, M.A.; Khatoon, F.; Ahmed, S. An overview on wounds their issue and natural remedies for wound healing. *Biochem. Physiol.* **2015**, *4*, 1453–1460. [2] Hussain, H.; Green, I.R.; Shamraiz, U.; Saleem, M.; Badshah, A.; Abbas, G.; Rehman, N.U.; Irshad, M. Therapeutic potential of glycyrrhetinic acids: A patent review (2010–2017). *Expert Opin. Ther. Pat.* **2018**, *28*, 383–398.

### 3.16. Microparticles Carrying Natural Antimicrobials for the Biological Control of Plant Diseases

CortesiRita^1^*MazzagliaAngelo^2^SguizzatoMaddalena^1^HallanSupandeep Singh^1^EspositoElisabetta^1^BalestraGiorgio M.^2^1Department of Life Sciences and Biotechnology (SVeB), University of Ferrara, 44121 Ferrara, Italy2Department of Agricultural and Forestry Science (DAFNE), University of Tuscia, 01100 Viterbo, Italy*Correspondence: crt@unife.it

It has been demonstrated that some aromatic substances synthesized by plants serve as plant defense mechanisms. However natural extracts are difficult to use due to their poor physico-chemical characteristics and low concentrations. In this study, two easily obtainable model compounds from plant tissues, namely gallic acid (GA) and ellagic acid (EA), were considered. Particularly, GA and EA were investigated for their employment as a support in the biocontrol of kiwifruit and/or tomato bacterial diseases aimed to reduce antibiotics and copper use that adversely affects the environment. Indeed, it is well known that over recent decades, as kiwifruit cultivation has gained increasing importance all over the world, unfortunately some bacterial diseases caused (for instance) by Pseudomonas syringae pv. actinidiae Takikawa et al., Pseudomonas syringae pv. syringae van Hall and Pseudomonas viridiflava (Burkholder) Dowson seriously threaten its cultivation. The same is true for tomato affected by Pseudomonas syringae pv. tomato (Pst). Our findings demonstrated that GA and EA are effective as pure substances in both in vitro and in vivo tests [1,2]. To overcome problems associated with antimicrobial physico-chemical characteristics, in the present study, natural and synthetic polymers such as hydroxyl-propyl-methylcellulose-phtalate (HPMCP), ethyl-cellulose (EC) and methyl-methacrylate (Eudragit RS 100) were used to produce spray-dried microparticles for the delivery of GA and EA. The selection of appropriate experimental parameters allowed us to obtain the standard conditions concerning instrumental settings, namely feed rate of polymer solution, air flow rate of the nebulization device, flow and temperature of drying air [1,2]. Optical and electron microscopies showed microparticles characterized by irregular morphologies and narrow size distribution, while HPLC studies demonstrated that microencapsulated GA and EA is stable and is not degraded. In vivo tests carried out in greenhouse tomato plants cv. Pullrex Bio contaminated with a bacterial suspension of Pst, 106 CFU/mL, demonstrated that HPMCP-GA microparticles were able to significantly reduce the Pst epiphytic population, showing an activity comparable to that obtained by copper salts. Moreover, when applied in greenhouse or in field on artificially and naturally infected kiwifruit plants, methyl-methacrylate microparticles showed an improvement and a remarkable prolongation of both GA and EA activity for up to 14 days after the treatment

The encouraging results obtained by these microencapsulated formulations allowed us to obtain innovative products which are useful to reduce phytotoxicity and which possibly point the way for future biological control strategies against bacterial diseases acting as alternatives to, or for use in combination with, low amounts of copper compounds.

**Acknowledgments:** Authors gratefully acknowledge Ministry of Agriculture, Food and Forestry of Italy MIPAAF for financial support (BBB PAN-2009). No funds were received to cover the costs to publish in open access.

**References:** [1] Cortesi, R.; Quattrucci, A.; Esposito, E.; Mazzaglia, A.; Balestra, G.M. Natural antimicrobials in spray-dried microparticles based on cellulose derivatives as potential eco-compatible agrochemicals. *J. Plant Dis. Prot.* **2017**, *124*, 269–278. [2] Rossetti, A.; Mazzaglia, A.; Muganu, M.; Paolocci, M.; Sguizzato, M.; Esposito, E.; Cortesi, R.; Balestra, G.M. Microparticles containing gallic and ellagic acids for the biological control of bacterial diseases of kiwifruit plants. *J. Plant Dis. Prot.* **2017**, *124*, 563–575.

### 3.17. Hydrophilic Sponges Loaded with Curcumin Solid Lipid Nanoparticles and Metronidazole Applied on L-PRF Clot to Promote Tissue Regeneration in Dentistry

MurgiaDenise^1^*MauceriRodolfo^1^ScialabbaCinzia^2^CampisiGiuseppina^1^De CaroViviana^2^1Dipartimento di Discipline Chirurgiche Oncologiche e Stomatologiche, Università degli Studi di Palermo 90133 Palermo, Italy2Dipartimento di Scienze e Tecnologie Biologiche Chimiche e Farmaceutiche (STEBICEF), Università degli Studi di Palermo, 90128 Palermo, Italy*Correspondence: denise.murgia@unipa.it

Leukocyte-and platelet-rich fibrin (L-PRF) technology make it possible to prepare strong fibrin membranes enriched with cells (activated platelets, leukocytes, circulating cells) and platelet growth factors [1]. In dentistry, the use of this autologous platelet concentrates such as L-PRF, also in association with Metronidazole (MTR), seems to present an innovative approach for vestibular bone grafting on the alveolar ridges [2]. In addition, Curcumin (CUR), a natural polyphenol derived from the rhizome of the Curcuma longa, could be effective on tissue regeneration due to its well-known antioxidant and anti-inflammatory properties [3]. The aim of the present work was the development of bioerodible sponges loaded with CUR-solid lipid nanoparticles (NLC) and MTR, assessing in vitro and ex vivo performance together with L-PRF, for future in vivo applications for tissues regeneration in oral surgeries. Curcumin (CUR)-loaded solid lipid nanoparticles (NLC) were prepared using Glycyrrhetic acid, hexadecanol, isopropyl palmitate and Tween 80 as surfactant. As a method, the homogenization followed by high-frequency sonication was used. After dialysis, NLC dispersion was evaluated in terms of drug loading (DL, 2.2% *w*/*w*) and recovery (DR, 88% *w*/*w*). NLC, characterized by Dynamic Light Scattering, exhibited an average particle size of 121.6 nm and PDI value of 0.235, considered optimal for a colloidal nanoparticle dispersion. The PZ value of −37 mV also indicated a good stability of the system. Subsequently, a hydrophilic sponge was obtained by lyophilization of a gel based on Trehalose, Natrosol, PVP, CUR-NLC dispersion and MTR (10% *w*/*w* of solid components). The ability of sponge to release CUR and MTR when applied on L-PRF clot, obtained according to the FDA and CE approved protocol (Intra-spin^®^, Intra-lock, Salerno, Italy), and the aptitude of actives to penetrate and/or permeate the membrane were evaluated. L-PRF clot was mounted as membrane in Franz type diffusion cells and to the apical side the sponges were applied. At the end of experiments, the residual drugs entrapped into the L-PRF membrane were quantified by extraction. The results showed that after 3 h, CUR is not able to cross the L-PRF clot, whereas a significant percentage of the dose (11.4%) remains trapped inside it. MTR cross L-PRF membrane and reaches the acceptor compartment gradually. After 3 h, the 16.8% of dose reaches plasma, whereas 6.5% was entrapped in the membrane.

In conclusion, both the CUR-NLC and the hydrophilic sponge containing MTR and CUR-NLC have been successfully prepared. When applied on L-PRF membrane, the sponges release the actives, promoting CUR penetration and MTR permeation. The obtained results encourage further studies about the possibility to use these sponges to deliver antioxidant and antimicrobial agents to support bone regeneration on surgical sites of teeth extraction treated with L-PRF.

**Acknowledgments:** D. Murgia, PhD student in Oncology and Experimental Surgery, Cycle XXXIII, is supported, for this research, by the MIUR (PON Industrial PhD 2017–2018—prot. DOT1320875).

**References:** [1] Del Corso, M.; Mazor, Z.; Rutkowski, J.L.; Ehrenfest, D.M.D. The use of leukocyte-and platelet-rich fibrin during immediate postextractive implantation and loading for the esthetic replacement of a fractured maxillary central incisor. *J. Oral Implantol.* **2012**, *38*, 181–187. [2] Simonpieri, A.; Del Corso, M.; Sammartino, G.; Dohan Ehrenfest, D.M. The relevance of Choukroun’s platelet-rich fibrin and metronidazole during complex maxillary rehabilitations using bone allograft. Part I: A new grafting protocol. *Implant Dent.* **2009**, *18*, 220–229. [3] Mouthuy, P.A.; Somogyi Škoc, M.; Čipak Gašparović, A.; Milković, L.; Carr, A.J.; Žarković, N. Investigating the use of curcumin-loaded electrospun filaments for soft tissue repair applications. *Int. J. Nanomed.* **2017**, *12*, 3977–3991.

### 3.18. Nanoparticles for Brain Disorders: From Formulative Study to In Vitro/In Vivo Behavior

EspositoElisabetta^1^*CortesiRita^1^DrechslerMarkus^2^FanJie^3^FuBingmei M.^3^CalderanLaura^4^BoschiFederico^4^NastruzziClaudio^1^1Department of Life Sciences and Biotechnology, University of Ferrara, I-44121 Ferrara, Italy2BIMF/Soft Matter Electronmicroscopy, University of Bayreuth, 95440 Bayreuth, Germany3Department of Biomedical Engineering City University of New York, New York, NY 1003, USA4Department of Neurological and Movement Sciences, University of Verona, 37134 Verona, Italy*Correspondence: ese@unife.it

Brain disorders such as neurodegenerative disease, brain tumors and traumatic brain injury represent dramatic conditions with extremely high unmet clinical needs. On this subject, many researchers are searching for strategies to safely and efficiently deliver biomolecules to the brain. For instance, it has been demonstrated that solid lipid nanomatrices can enhance drug delivery to the brain. In this regard, in the present investigation, solid lipid nanoparticles (SLN) have been considered as tools to treat neurological disorders, such as multiple sclerosis and post-traumatic stress. Particularly dimethyl fumarate (DMF) or cannabinoid drugs (CD) (i.e., URB597, AM251 and rimonabant) containing SLN have been produced and characterized. Indeed, DMF is useful in relapsing remitting multiple sclerosis treatment, while CD can treat post-traumatic stress, anxiety, depression and neurodegenerative disorders such as multiple sclerosis. Namely, SLN based on tristearin and nanostructured lipid carriers (NLC), constituted of tristearin in combination with the liquid caprylic/capric triglyceride, were investigated. Different strategies have been adopted to obtain nanoparticles that are able to reach the brain. For instance, SLN and NLC were treated with polysorbate 80, while cationic SLN were obtained employing mixture of tristearin and dimethyldioctadecylammonium chloride. The morphology and dimensional distribution of nanoparticles have been investigated by cryogenic transmission electron microscopy and photon correlation spectroscopy, while drug encapsulation efficiency was evaluated by HPLC analysis. Notably, to investigate in vitro permeability and in vivo biodistribution in mice, fluorescent nanoparticles were produced and characterized. Cationic SLN exhibited higher permeability values with respect to neutral SLN and SLN treated by polysorbate 80, as demonstrated by an in vitro permeability study conducted through a model of mouse brain microvascular endothelial cells. Fluorescent luminescence imaging enabled us to investigate the biodistribution of polysorbate 80 treated SLN after intraperitoneal or intranasal administration in mice. Even though in vivo images evidenced a prevalent accumulation of polysorbate 80 treated SLN in liver and spleen, fluorescence was also impressively found in the brain, suggesting the possible suitability of SLN to treat brain disorders.

**Acknowledgments:** This work was funded by “FIRB 2010 from the Ministry of the University and Research of Italy (code RBFR10XKHS). We did not received funds for covering the costs to publish in open access.

### 3.19. Drug in Micelles in Deformable Liposomes (DiMiLs) as a Novel Delivery System for Poor Permeable Hydrophobic Drugs

FranzéSilvia*MinghettiPaolaCilurzoFrancescoDepartment of Pharmaceutical Sciences, Università degli Studi di Milano, via G. Colombo 71, 20133 Milan, Italy*Correspondence: silvia.franze@unimi.it

Deformable lipid vesicles are a subject of great interest as (trans)dermal drug delivery systems for their ability to penetrate the stratum corneum barrier. This property is mainly due to the “soft” structure of these nanocarriers which arises from the presence in the bilayer of a destabilizing agent. Nevertheless, the low packing order of the bilayer is responsible for the poor stability of vesicles, with a high extent of drug leakage over time. This phenomenon is emphasized in the case of hydrophobic compounds that may freely diffuse through the mobile lipid chains towards the bulk solution [1]. In this work, a novel drug in micelles in the deformable liposomes (DiMiLs) system is proposed to prevent the leakage of hydrophobic compounds from soft carriers.

Basically, kolliphor HS 15 micelles were used to solubilize hydrophobic model compounds (nifedipine and piroxicam) in the aqueous core of deformable liposomes (DL) composed of egg-phosphatidylcholine and Tween 80 in different weight ratios (85:15 or 95:5 *w*/*w*). The performances of DiMiLs and DL were compared in terms of drug release, deformability, physico-chemical stability and skin permeation. The encapsulation of the drug in micelles significantly reduced the concentration gradient towards the bulk medium and, as a result, slowed the drug release kinetic of DiMiLs when compared to DL. In accordance with this, DiMiLs allowed us to reduce or abolish drug leakage. In fact, the drug leakage from DL was high after only one month of storage (almost 50% in the case of nifedipine and in the range of 39–79% in the case of piroxicam loaded DL, depending on T80 content). Optimized DiMiL formulations instead retained the drug content for up to two-months of storage. The encapsulation of surfactant-based micelles in the core of DL did not compromise the deformability of the carriers. All DiMiLs formulations showed values of constant of deformability (k) comprised within 0.01 and 0.05 N/mm and then suitable for (trans)dermal administration [2]. Surprisingly, the DiMiLs significantly increased the permeation of both model drugs through human skin with respect to DL (of about 48 and 4 times for nifedipine and piroxicam, respectively).

In conclusion, the proposed system makes it possible to stabilize soft vesicles without compromising their deformability. DiMiLs also reveal a great ability to enhance the permeation of poor permeable drugs through human skin, and therefore, they are worth of further investigation as transdermal drug delivery systems.

**References:** [1] Franzé, S.; Marengo, A.; Stella, B.; Minghetti, P.; Arpicco, S.; Cilurzo, F. Hyaluronan-decorated liposomes as drug delivery systems for cutaneous administration. *Int. J. Pharm.* **2018**, *535*, 333–339. [2] Franzé, S.; Donadoni, G.; Podestà, A.; Procacci, P.; Orioli, M.; Carini, M.; Minghetti, P.; Cilurzo, F. Tuning the extent and depth of penetration of flexible liposomes in human skin. *Mol. Pharm.* **2017**, *14*, 1998−2009.

### 3.20. inPentasomes: A N2B approach to MPTP Parkinsonism in Mice

RinaldiF.^1^SeguellaL.^2^GigliS.^2^HaniehP.N.^3^*ImbrianoA.^3^Del FaveroE.^4^CantùL.^4^PesceM.^5^SarnelliG.^5^MarianecciC.^3^EspositoG.^2^CarafaM.^3^1Fondazione Istituto Italiano di Tecnologia, Center for Life Nano Science@Sapienza, 00185 Rome, Italy2Department of Physiology and Pharmacology “V. Erspamer”, Sapienza University of Rome, 00185 Rome, Italy3Department of Drug Chemistry and Technology, University of Rome “Sapienza”, 00185 Rome, Italy4Department of Medical Biotechnologies and Translational Medicine, University of Milan, 20133 Milan, Italy5Department of Clinical Medicine and Surgery, University of Naples ‘Federico II’, 80131 Naples, Italy*Correspondence: patrizianadia.hanieh@uniroma1.it

Preclinical and clinical evidence has demonstrated that astroglial-derived S100B protein is a key element in neuroinflammation underlying the pathogenesis of Parkinson’s disease (PD), in so much as that S100B inhibitors have been proposed as promising candidates for PD targeted therapy. Pentamidine, an old antiprotozoal drug which is currently used for pneumocystis carinii, is one of the most potent inhibitors of S100B activity; however, despite this effect, it is limited by its low capability to cross blood brain barrier (BBB). To overcome this problem, we developed a noninvasive intranasal delivery system, chitosan coated niosomes with entrapped pentamidine (inPentasomes), in the attempt to provide a novel pharmacological approach to ameliorate parkinsonism induced by subchronic MPTP administration in C57BL-6J mice. inPentasomes, prepared by an evaporation method, were administered daily by intranasal route in subchronic MPTPintoxicated rodents and resulted in a dose-dependent manner (0.001–0.004 mg/kg) capable for a significant Tyrosine Hydroxylase (TH) positive neuronal density rescue in both striatum and substantia nigra of parkinsonian mice. In parallel, inPentasomes significantly decreased the extent of glial-related neuroinflammation through the reduction of specific gliotic markers (Iba-1, GFAP, COX-2, iNOS) with consequent PGE2 and NO2—release reduction, in nigrostriatal system. inPentasomes-mediated S100B inhibition resulted in a RAGE/NF-κB pathway downstream inhibition in the nigrostriatal circuit, causing a marked amelioration of motor performance in intoxicated mice. On the basis of our results, chitosan coated niosomes loaded with pentamidine, the inPentasome system, self-candidates as a promising new intranasal approach to mitigate parkinsonism in humans, and possibly paves the way for a possible clinical repositioning of pentamidine as anti-PD drug. InPentasomes inhibit MPTP-induced dopaminergic neuronal loss in nigrostriatal areas.

**Acknowledgments:** This work was supported by Sapienza Ateneo funding “Multidisciplinari 2015” C26M15SP9F.

### 3.21. Glycogen Cationic Nanovectors Potentially Useful for siRNA Delivery

RacanielloG. F.^1^LopedotaA.^1^FrancoM.^1^CutrignelliA.^1^LaquintanaV.^1^LopalcoA.^1^LiberatiE.^2^RussoV.^2^DenoraN.^1^*1Department of Farmacia—Scienze del Farmaco, University of Bari “Aldo Moro”, 70121 Bari, Italy2ACRAF Angelini Research Centre, 00071 Pomezia, Italy*Correspondence: nunzio.denora@uniba.it

Polglumyt is a highly purified form of glycogen, characterized by high water solubility (30% *w*/*v*), which is accompanied by a very moderate increase in viscosity due to the hyperbranched nature of the macromolecule. Its dendrimeric structure, appropriately functionalized, makes it an alternative to current synthetic gene delivery agents such as dendrimers or hyperbranched polymers [1]. Polglumyt Policationic Derivates (PPDs) are hyperbranched nanocarriers for nucleic acids, which also combine natural origin, degradability, negligible toxicity and good loading capacity. PPDs are synthesized starting from a purified form of glycogen by introducing cationic groups into polymer chains, which can electrostatically interact with nucleic acids. The present work describes the preparation of PPDs, and their characterization. PPDs were synthesized in different N,N-dialkylaminoalkyl halides/Polglumyt molar ratios under alkaline aqueous conditions. All derivatives were characterized by nuclear magnetic resonance, gel permeation chromatography and shear rheometry. PPDs were synthetized in good to excellent yields (60–80%). DLS was used to measure hydrodynamic size, size distribution and to verify the presence of aggregation phenomena. All size values are in a range between 20 and 70 nm with a PDI lower than 0.3. Measurements for zeta-potential were performed by LDV technique and zeta-potential values show an increase in relation to concentration of *N*,*N*-dialkylaminoalkyl halides passing from −9 to +45 mV. The PPDs were also characterized regarding their pKa values, titrating PPDs aqueous solution with a 1 N NaOH solution. All PPDs showed a similar value of pKa, i.e., in range of 11.7. All derivatives are characterized by the presence of two different basic groups: the first has a pKa above the physiological pH, which should guarantee binding with nucleic acids, whereas the second should promote endosomal escape by the proton sponge effect. By optimizing Polglumyt derivatives’ structures in terms of the extent of derivatization and nature of the cationic groups, we could achieve high RNA loadings with negligible aggregation, and good protection from nuclease degradation with negligible cytotoxicity.

**References:** [1] Perrone, M.; Lopedota, A.; Liberati, E.; Russo, V.; Cutrignelli, A.; Laquintana, V.; de Sousa, I.P.; Franco, M.; Tongiani, S.; Denora, N.; et al. Natural dendrimers: Synthesis and in vitro characterization of glycogen-cysteamine conjugates. *Eur. J. Pharm. Biopharm.* **2017**, *115*, 168–176.

### 3.22. Photodynamic Active Substances and Their Use for Photodynamic Therapy of the Tumor

ŽárskáLudmila*MaláZuzanaKolářováHanaDepartment of Medical Biophysics, Faculty of Medicine and Dentistry, Palacky University, Hněvotínská 3, 775 15 Olomouc, Czech Republic*Correspondence: ludmila.zarska@centrum.cz

Photodynamic therapy (PDT) is currently another option for the treatment of tumors. This therapy is a combination of a photodynamically-active substance and light of a suitable wavelength. Two tumor cell cultures—HeLa and G361 were used for the in vitro study. Cells were cultured in 96-well plates with TMPyP and ZnTPPS4 photosensitizers at concentrations of 0.25 μM, 0.5 μM and 5 μM. As a light source, a diode emitter with a wavelength of 414 nm and a radiation intensity of 5 Jcm^−2^, 25 Jcm^−2^ was used and at irradiation times of 1 min 33 s, 7min 43 s. In this study, three measurement methods were used—a viability test (MTT), determination of ROS production and monitoring of changes in membrane potential (JC1). ROS detection showed that both cell cultures showed a more pronounced increase in use TMPyP photosensitizer at a concentration of 5 μM and a radiation intensity of 25 Jcm^−2^. The smallest effect on cell cultures was ZnTPPS4, with a concentration of 0.25 μM and a radiation intensity of 5 Jcm^−2^. The MTT test demonstrated a higher efficacy of the TMPyP photosensitizer compared to ZnTPPS4. Changes in membrane potential have been demonstrated primarily in relation to irradiation parameters. A suitable combination of photosensitizer and radiation may be an effective therapy for the treatment of cancer.

**Acknowledgments:** The thesis was developed with the support of Study of cellular signaling in relation to illness IV (LF2017021).

### 3.23. Silk Fibroin Nanoparticles for Celecoxib and Curcumin Delivery: In Vitro Efficacy in an Osteoarthritis Model

PerteghellaSara^1^,^2^*BariElia^1^CrivelliBarbara^1^CatenacciLaura^1^SorrentiMilena^1^MocchiMichela^1^,^3^TripodoGiuseppe^1^Prina-MelloAdriele^3^TorreMaria Luisa^1^,^2^1Department of Drug Sciences, University of Pavia, 27100 Pavia, Italy2PharmaExceed srl, 27100 Pavia, Italy3Laboratory of Biological Characterization of Advances Materials, Trinity Translational Medicine Institute, Trinity College Dublin, The University of Dublin, Dublin 2, Ireland*Correspondence: sara.perteghella@unipv.it

Osteoarthritis (OA) represents a musculoskeletal pathological condition characterized by chondrocytes apoptosis, extracellular matrix degradation and consequent cartilage degeneration; the main mechanisms involved in OA onset seem to be oxidative stress and the overproduction of pro-inflammatory cytokines, such as interleukin-1β (IL-1β) and tumour necrosis factor-α (TNF-α) [1,2]. Systemic therapy with anti-inflammatory drugs represents the “gold standard” in OA treatment, and Celecoxib is the first choice. However, it presents limited water solubility, poor in vivo bioavailability, fast metabolism and bloodstream clearance^2^. Nanomedicine is considered a good strategy to overcome these problems; in fact, nanosystems are able to improve the solubility and the bioavailability of poorly-soluble drugs, and to control/target the drug release, avoiding undesired side effects. Thanks to its biocompatibility and biodegradability, silk fibroin represents the ideal biomaterial candidate for the nanoparticle production [3]. In this study, we selected silk fibroin nanoparticles (SFNs) as the delivery system for two anti-inflammatory hydrophobic drugs: celecoxib (CXB) and curcumin (CUR). Both empty and loaded nanoparticles were prepared by desolvation method, and physico-chemically characterized (FT-IR, DSC, TGA, SEM, particle size distribution and in vitro drug release). The safety and efficacy of nanosystems were determined in terms of hemolytic properties, cytotoxicity, ROS-scavenging activity and anti-inflammatory potency in an OA in vitro cell model. In particular, chondrocytes were stimulated with IL-1β to simulate an inflammatory condition, and treated with nanoparticles or free drugs. As output data, we considered the cell secretion of cytokines and chemokines (Interleukin-6: IL-6; Regulated on Activation, Normal T cell Expressed and Secreted: RANTES). Results indicate that a controlled drug release has been achieved by varying the drug loading. A synergistic antioxidant effect of curcumin and fibroin (SFNs-CUR) was demonstrated by the DPPH assay while CXB resulted, in some manner, inhibitory. Free CUR and CXB were shown to be highly cytotoxic on in vitro cultured chondrocytes; however, high cell viability data were obtained by treating chondrocytes with encapsulated drugs, demonstrating the protective effect of developed nanoparticles. The release of pro-inflammatory factors, IL-6 and RANTES, by inflamed chondrocytes was significantly reduced by treating the cells with all considered nanoparticles (unloaded and CUR or CXB-loaded). Promising results were obtained with fibroin nanoparticles loaded with curcumin, which presented the same in vitro anti-inflammatory activity with respect to free CXB. In conclusion, silk fibroin nanoparticles can be considered as optimal carriers to improve the compatibility profile of CUR and CXB; on the other hand, SFNs were demonstrated to have intrinsic anti-inflammatory activity and a synergic effect when used as vehicle for CUR. Furthermore, SFNs allowed us to control the time of drug release while maintaining the dose within the expected delivery window. This supports the explained biological effects seen over time, and enables drug release designing from SFNs by controlling the amount of the loaded drug.

**References:** [1] Alaaeddine, N.; Olee, T.; Hashimoto, S.; Creighton-Achermann, L.; Lotz, M. Production of the chemokine RANTES by articular chondrocytes and role in cartilage degradation. *Arthritis Rheum.* **2001**, *44*, 1633–1643. [2] Paulson, S.K.; Vaughn, M.B.; Jessen, S.M.; Lawal, Y.; Gresk, C.J.; Yan, B.; Maziasz, T.J.; Cook, C.S.; Karim, A. Pharmacokinetics of celecoxib after oral administration in dogs and humans: effect of food and site of absorption. *J. Pharmacol. Exp. Ther.* **2001**, *297*, 638–645. [3] Perteghella, S.; Crivelli, B.; Catenacci, L.; Sorrenti, M.; Bruni, G.; Necchi, V.; Vigani, B.; Sorlini, M.; LuisaTorre, M.; Chlapanidas, T. Stem cell-extracellular vesicles as drug delivery systems: New frontiers for silk/curcumin nanoparticles. *Int. J. Pharm.* **2017**, *520*, 86–97.

### 3.24. Newly Synthesized Penetratin-Derived Decapeptides: Ex Vivo Permeation across Porcine Cornea

PescinaSilvia^1^*SalaMarina^2^ScalaMaria Carmina^2^SalernoKetty^1^PadulaCristina^1^SantiPatrizia^1^OstacoloCarmine^3^NicoliSara^1^1Food and Drug Department, Univ. of Parma, Parco Area delle Scienze 27/a, 43124 Parma, Italy2Department of Pharmacy, Univ. of Salerno, via G. Paolo II 131, 84084 Fisciano, Salerno, Italy3Department of Pharmacy, Univ. of Naples Federico II, via D. Montesano 49, 80131 Napoli, Italy*Correspondence: silvia.pescina@unipr.it

Cell penetrating peptides (CPPs) are a family of natural and synthetic cationic peptides (5–40 amino acids), known for their ability to be easily internalized by cells. For this reason, they have been widely studied in the field of drug delivery as possible drug carriers. Recently, interest in CPPs has also involved ocular administration, since these peptides represent a potential powerful tool for the treatment of diseases affecting both the anterior and the posterior segments of eye [1]. Previously, we reported the synthesis and ex vivo evaluation of some derivatives of PEP-1, a well-known CPP [2]; the aim of the present work was to investigate the ability of newly-synthesized, penetratin-derived decapeptides to entirely cross the isolated porcine cornea. Decapeptides were obtain following a mimotopic approach, starting from penetratin sequence (PNT: RQIKIWFQNRRMKWKK), recognized as amodel CPP and considered as a reference. First, two analogues of PNT were prepared by adding a spacer, consisting of two glycine residues (GG) between the aminoacidic sequence and the fluorescent label (5-carboxyfluorescein; FAM), respectively to the arginine (R) residue (PNT_arg_: FAM-GGRQIKIWFQNRRMKWKK) or to the lysine (K) residue (PNT_lys_: FAM-GGKKWKMRRNQFWIKIQR) of the PNT sequence. Then, starting from PNT_arg_ and PNT_lys_ and following a mimotopic approach, ten fluorescently-labeled decapeptides were designed and synthesized (PNT_arg_1-5 and PNT_lys_1-5). To assess their ex vivo corneal permeability, permeation experiments were carried out using fresh and whole porcine corneas mounted on Franz cells. Preliminary data confirmed the ability of peptides to cross all three corneal layers, despite the relatively high molecular weight (*approx.* 1.5–2.8 kDa). In fact, after 6 h of experiments, detectable amounts of CPPs were found inside the receiving chamber. The presence of GG spacer within PNT_arg_ apparently did not influence its diffusion with respect to PNT; at the same time, the inversion of the amino acid sequence (PNT_arg_ vs. PNT_lys_) did not affect the behavior. In general, by reducing the number of amino acids (decapeptides), no evidence of improvement in the trans-corneal transport was observed, since the permeability coefficients were lower than PNT (i.e., PNT_lys_3 was three orders of magnitude less than PNT) or comparable (i.e., PNT_arg_1). It is necessary to underline that the permeation data are affected by a relevant variability that could be ascribed to the propensity of CPPs to enter corneal epithelial and endothelial cells. To verify this hypothesis and to check also the safety of the CPPs, further investigations such as cellular uptake and cytotoxicity experiments are ongoing.

**References:** [1] Pescina, S.; Ostacolo, C.; Gomez-Monterrey, I.M.; Sala, M.; Bertamino, A.; Sonvico, F.; Padula, C.; Santi, P.; Bianchera, A.; Nicoli, S. Cell penetrating peptides in ocular drug delivery: State of the art. *J. Control. Release* **2018**, *284*, 84–102. [2] Pescina, S.; Sala, M.; Padula, C.; Scala, M.C.; Spensiero, A.; Belletti, S.; Gatti, R.; Novellino, E.; Campiglia, P.; Santi, P.; et al. Design and Synthesis of New Cell Penetrating Peptides: Diffusion and Distribution Inside the Cornea. *Mol. Pharm*. **2016**, *13*, 3876–3883.

### 3.25. α-Tocopherol Nanoemulsions as Transporters across the Caco-2 Enterocyte-Like Model

Plaza-OliverM.^1^,^2^*BeloquiA.^3^Santander-OrtegaM.J.^1^,^2^Rodríguez-RobledoV.^1^,^2^Castro-VázquezL.^1^,^2^González-FuentesJ.^1^,^2^MarcosP.^1^,^2^Arroyo-JiménezM.M.^1^,^2^LozanoV.^1^,^2^PréatV.^3^1Cellular Neurobiology and Molecular Chemistry of the Central Nervous System Group, Faculty of Pharmacy, University of Castilla-La Mancha, 02008 Albacete, Spain2Regional Centre of Biomedical Research (CRIB), University of Castilla-La Mancha, 02008 Albacete, Spain3Louvain Drug Research Institute, Advanced Drug Delivery and Biomaterials, Université Catholique de Louvain, 1200 Brussels, Belgium*Correspondence: maria.plaza3@alu.uclm.es

For the oral administration of drugs to succeed requires the drug to overcome the harsh gastrointestinal environment and the intestinal barrier itself, constituted mainly by the intestinal monolayer and a thick cover of mucus [1]. The development of colloidal drug delivery systems has greatly contributed to improving the bioavailability of labile, poorly-soluble and low permeable drugs [1]. Among colloids, nanoemulsions are attractive vesicular nanosystems whose core-surface structures can be adjusted due to their versatility [2]. This helps to modulate their in vivo behavior and to adjust the properties of the system to the specific requirements of the administration route. We have previously observed that the thorough selection of the interface composition led to higher α-tocopherol bioaccesibility levels when decreasing the amount of lecithin at the surface in α-tocopherol constituted nanoemulsions [3]. The aim of this study was to evaluate the in vitro performance of α-tocopherol nanoemulsions in the well-stablished Caco-2 intestinal model. The physicochemical characterization of the nanoemulsions showed size values of 170 nm, barely polydispersed and negatively charged. The cytotoxicity studies confirmed the absence of toxicity after two hours of co-incubation with Caco-2 cells, and unaltered trans-epithelial electrical resistance (TEER) values after this period. The ability of the nanoemulsions to translocate across the monolayer was determined by monitoring the amount of α-tocopherol in the basolateral chamber. This approach makes possible the direct quantification of a molecule that constitutes the system itself by using a high-performance liquid chromatography (HPLC) as analytical method. The nanoemulsions showed a good transport rate (P_app_ = 5.46 × 10^−6^ ± 1.11 × 10^−6^ cm/s), indicating that the systems were effectively transported across the epithelium. These results prove (i) that α-tocopherol nanoemulsions translocate through the monolayer, and (ii) that the quantification of the nanosystem transport per se is feasible without the use of a fluorescent moiety within the systems. This represents a remarkable advantage over current drug delivery strategies intended for the oral route of administration.

**Acknowledgments:** M. Plaza thanks the financial support given by the UCLM (OE154), Spain. A. Beloqui is a Research Associate of the *Fonds de la Recherche Scientifique-FNRS* (Belgium).

**References:** [1] Date, A.A.; Hanes, J.; Ensign, L.M. Nanoparticles for oral delivery: Design, evaluation and state-of-the-art. *J. Control. Release* **2016**, *240*, 504–526. [2] Santander-Ortega, M.J.; Plaza-Oliver, M.; Rodríguez-Robledo, V.; Castro-Vázquez, L.; Villaseca-González, N.; González Fuentes, J.; Cano, E.; Marcos, P.; Lozano, M.V.; Arroyo Jiménez, M.M. PEGylated nanoemulsions for oral delivery: role of the inner core on the final fate of the formulation. *Langmuir* **2017**, *33*, 4269–4279. [3] Plaza-Oliver, M.; Baranda, J.F.; Rodríguez-Robledo, V.; Castro-Vázquez, L.; González Fuentes, J.; Marcos, P.; Lozano, M.V.; Santander-Ortega, M.J.; Arroyo Jiménez, M.M. Design of the interface of edible nanoemulsions to modulate the bioaccessibility of neuroprotective antioxidants. *Int. J. Pharm.* **2015**, *490*, 209–218.

### 3.26. Jet Injection of Polymeric Microparticles: Role of Size and Stiffness on Particle Delivery through the Skin

PrimaveraRosita^1^SchlichMichele^1^LaiFrancesco^2^FaddaAnna Maria^2^SinicoChiara^2^DecuzziPaolo^1^*1Laboratory of Nanotechnology for Precision Medicine, Italian Institute of Technology, 16163 Genova, Italy2Dept. of Life and Environmental Sciences, University of Cagliari, 09124 Cagliari, Italy*Correspondence: paolo.decuzzi@iit.it

Needle-free jet injectors are devices that use a high-speed stream of fluid to puncture the skin and deliver medications in the sub-cutaneous/intramuscular regions, eliminating the risk of accidental needle-stick injuries and offering a pain-less alternative to people suffering with needle phobia. These devices are currently used in clinical practice for mass immunization and the delivery of pharmaceutical solutions of macromolecules (such as insulin and growth hormones), as well as small molecules including antibiotics, local anesthetics and steroids [1]. Recently, the feasibility of using jet injectors to administer nanoparticulate drug delivery systems was evaluated, broadening the range of applications of such medical devices [2]. In this work, curcumin-loaded, poly-lactide-co-glycolide (PLGA) microplates (μPLs), and also discoidal polymeric nanoparticles (DPNs) were prepared using a top-down fabrication approach, which allowed us to tune their mechanical and geometrical properties [3,4]. Specifically, μPLs have a square base with an edge length of 20 μm and a variable height (5–10 µm), while DPNs are characterized by a discoidal shape with a diameter of 2 μm and height of 0.6 μm. The shape, size, surface properties, mechanical stiffness and biodegradability of the developed particles make them ideal for tissue implantation and prolonged release of active molecules and imaging contrast agents. The combined use of a commercial needle-free jet injector with μPLs and DPNs, is explored with the objective of realizing a sub-cutaneous drug depot with high patients’ compliance. To this end, μPLs of different size and stiffness, and DPNs were injected through a porcine skin specimen. The particle number, shape and integrity were assessed before and after the injection by multisizer particle counter and scanning electron microscopy. Results showed no detrimental effect on particle morphology upon high velocity impact with the skin, although the formulations prepared with lower amount of polymer were more prone to remain stuck within the epidermal network. These findings are supported by HPLC data, which showed a higher amount of penetrated curcumin when polymer-rich microparticles were injected. Collectively, these data encourage the future development of this combined approach, which would be particularly beneficial for the administration of medications requiring a prolonged action, such as antipsychotics and macromolecules for metabolic diseases.

**Acknowledgments:** This project was partially supported by the European Research Council, under European Union’s Seventh Framework Programme (FP7/2007–2013)/ERC grant agreement no 616695, by the Italian Association for Cancer Research (AIRC) under the individual investigator grant No 17664, and by the European Union’s Horizon 2020research and innovation program under the Marie Sklodowska-Curie grant agreement No 754490. The authors acknowledge the precious support provided by the Electron Microscopy and Nanofabrication facilities at the Italian Institute of Technology.

**References:** [1] Mitragotri, S. Current status and future prospects of needle-free liquid jet injectors. *Nat. Rev. Drug Discov.* **2006**, *5*, 543–548. [2] Schlich, M.; Lai, F.; Murgia, S.; Valenti, D.; Fadda, A.M.; Sinico, C. Needle-free jet injection of intact phospholipid vesicles across the skin: A feasibility study. *Biomed. Microdevices* **2016**, *18*, 67. [3] Di Francesco, M.; Primavera, R.; Romanelli, D.; Palomba, R.; Pereira, R.C.; Catelani, T.; Celia, C.; Di Marzio, L.; Fresta, M.; Di Mascolo, D.; et al. Hierarchical Microplates as Drug Depots with Controlled Geometry, Rigidity, and Therapeutic Efficacy. *ACS Appl. Mater. Interfaces* **2018**, *10*, 9280–9289. [4] Palomba, R.; Palange, A.L.; Rizzuti, I.F.; Ferreira, M.; Cervadoro, A.; Barbato, M.G.; Canale, C.; Decuzzi, P. Modulating Phagocytic Cell Sequestration by Tailoring Nanoconstruct Softness. *ACS Nano* **2018**, *12*, 1433–1444.

### 3.27. Multi-Drug Local Delivery System for Glioblastoma through Lauroyl-Gemcitabine Nanomedicine Hydrogel

BozzatoElia^1^BastiancichChiara^2^PréatVéronique^1^*1Louvain Drug Research Institute, Advanced Drug Delivery and Biomaterials, Université Catholique de Louvain, 1200 Brussels, Belgium2Institut de NeuroPhysiopathologie—CNRS, UMR 7051, Aix-Marseille Université, 13344 Marseille, France*Correspondence: veronique.preat@uclouvain.be

Glioblastoma (GBM) is the most common and aggressive brain tumor in adults. The first line treatment for this disease is surgical resection of the main tumor mass followed by radiotherapy and concomitant treatment with temozolomide. However, due to the aggressiveness and rapid proliferation of the tumor, patients’ survival with this gold standard treatment is only slightly extended (median survival 14 months). Surgical resection is often limited to avoid neurological and cognitive damages. Moreover, cancer stem cells, which are a subpopulation of the tumor cells that possess high self-renewing and tumorigenic capabilities, present intrinsic radio- and chemoresistance. For these reasons, recurrences arising along the resection cavity are the most common and inevitable consequence of this treatment. The aim of this project is to avoid the onset of tumor recurrences by a Lauroyl-gemcitabine (GemC12)-loaded injectable hydrogel that can be injected in the resection cavity. This makes it possible to bypass all the CNS barriers, and to exploit the “empty” core of the nanoparticles to load a second drug that acts on cancer stem cells by interfering with the molecular pathways that makes them different from normal tumor cells. The gel, already developed in our laboratory, consists of GemC12-loaded lipid nanocapsules (LNCs), where the drug also acts as crosslinking agent between the nanocapsules themselves, thus forming a network that can entrap water molecules. The gel prepared through a phase-inversion technique presents a sustained release of the drug and mechanical properties compatible for brain implantation. Moreover, the in vivo efficacy of the system leads to a significant delay of tumor growth compared to the treatment with Gemcitabine, thus showing encouraging characteristics for the application in the biopharmaceutical field in terms of stability, safety and efficacy. In this work, we aim at combining GemC12 with drugs able to act on glioma stem cells. In vitro studies on two GBM cell lines (U87MG and 9L) have shown a synergic effect between GemC12 and Salinomycin and GemC12 and Curcumin. On the other side, no synergy was demonstrated with GemC12 and Metformin and GemC12 and Resveratrol. Thanks to the synergy between GemC12 and the second drug encapsulated in the core of the nanocapsules, it will be possible to obtain a sustained release of the two drugs, a higher cytotoxic effect both on normal cancer cells and cancer stem cells, and an increase in the efficacy of the drug delivery system, thus potentially leading to the complete eradication of the tumor.

**Acknowledgments:** Gliogel project is funded by EuroNanoMedIII.

### 3.28. Mannose-Targeted Cationic Glycopolymers as New Tool for Nucleic Acid-Based Cancer Immunotherapy

BellatoFedericaBrunatoSilviaMaglioccaSalvatoreRomanoCristianaAvanciniGretaCalicetiPaoloSalmasoStefanoMastrottoFrancesca*Department of Pharmaceutical and Pharmacological Sciences, University of Padua, Via F. Marzolo 5, 35131 Padova, Italy*Correspondence: francesca.mastrotto@unipd.it

The delivery of pDNA or mRNA coding for Tumor Associated Antigens (TAA) is emerging as a new strategy for anticancer vaccination. To date, lipid-based systems and viral vectors dominate the nucleic acid delivery scenario. However, synthetic polymeric carriers have recently drawn increasing attention. Here, Reversible Addition Fragmentation chain Transfer (RAFT) polymerization [1] has been exploited for the synthesis of a small library of diblock copolymers designed to deliver nucleic acids encoding tumor-associated antigen (TAA) to dendritic cells and to trigger the immune response and memory against cancer. These novel materials were designed with a poly-cationic agmatine block to condense nucleic acids and with a glycopolymeric block to actively target Mannose Receptor (MR) on immune cells [2]. Importantly, the system is also expected to provide nucleic acid protection against fast degradation, minimizing its interactions with extracellular nucleases. Starting from Agmatine acrylamide (Agm) and D-Mannose acrylamide (Man) monomers, three cationic block copolymers (Man15-*b*-Agm12, Man29-*b*-Agm25 and Man58-*b*-Agm45) were generated by fast RAFT polymerization. Polymers with same monomer feed ratio and increasing lengths were synthesized to select the one that ensured suitable loading, stability, delivery. GPC analysis on glycopolymers confirmed their narrow molecular weight distribution, with a polydispersity of 1.13, 1.43 and 1.29, respectively. Glycopolyplexes (GPPs) were obtained by incubating each block co-polymer with a 19-base ssDNA, used as model oligonucleotide. The optimum N/P ratio to achieve complete ssDNA complexation was evaluated by gel electrophoresis. A complete DNA complexation occurred also in presence of physiological concentration of heparin at N/P ratios higher than 20, 10 and 5 for Man15-*b*-Agm12, Man29-*b*-Agm25 and Man58-*b*-Agm45, respectively. Furthermore, DLS and TEM characterization confirmed the formation of GPPs with narrow size distribution in the range of 25-45 nm, depending on the polymer size. Finally, flow cytometric studies revealed a remarkably high and specific recognition by mannose receptor expressing cells (CHO-MR) for Man15-*b*-Agm12 and Man29-*b*-Agm25 GPPs with negligible internalization by cells that do not express the receptor (CHO). Man58-*b*-Agm45 polyplexes showed 2-fold higher internalization in CHO-MR cells as compared to control cell line. Ongoing studies will investigate the polymers’ suitability for pDNA complexation and delivery, thus enabling their exploitability for cell transfection and anticancer vaccination.

**References:** [1] Graeme, M.; Rizzardo, E.; Thang, S. Radical addition–fragmentation chemistry in polymer synthesis. *Polymer* **2008**, *49*, 1079–1131. [2] Tibor, K.; Ramakrishna, V.; Fanger, M. Mannose receptor-targeted vaccines. *Expert Opin. Biol. Ther.* **2004**, *4*, 1953–1962.

### 3.29. Dexamethasone Loaded Oligo-Cationic Liposomes in the Posterior Segement Ocular Delivery

MdAl-Amin^1^BressanAlice^1^BalassoAnna^1^MastrottoFrancesca^1^UrttiArto^2^CalicetiPaolo^1^SalmasoStefano^1^*1Department of Pharmaceutical and Pharmacological Sciences, University of Padova, Via F. Marzolo 5, 35131 Padova, Italy2Division of Pharmaceutical Biosciences, University of Helsinki, Viikinkaari 5 E, 00014 Helsinki, Finland*Correspondence: stefano.salmaso@unipd.it

Age-related macular degeneration, diabetic retinopathy, glaucoma, retinal degeneration are the most represented posterior segment ocular diseases [1]. The progress of these diseases is characterized by onset of inflammation affecting retinal cells [2]. Delivering therapeutic molecules to the eye has always been a challenge because of the involvement of various physiological barriers, e.g., inner limiting membrane (ILM) [3]. Liposomes represent a valid strategy to improve residence time of drugs in the vitreous, thus reducing administration frequency, and to protect unstable drugs from degradation. Here, we aimed at modulating the surface properties of liposomes with a combination of mPEG2 kDa-DSPE and a newly-synthesized, oligocationic, non-peptidic, non-linear cell penetration enhancer (CPE) to control both their diffusivity in the vitreous and their intracellular access. The nano platform has been used to deliver the anti-inflammatory agent dexamethasone by intravitreal administration. HSPC/Cholesterol liposomes were prepared by rehydration with calcium acetate and were remotely loaded with dexamehasone hemisuccinate by exploiting the high calcium acetate gradient. A variety of formulation parameters were investigated to assess the effect on the loading efficiency and capacity: calcium acetate concentration, medium pH, osmotic agent, phospholipid to drug ratio, incubation time, lipid composition and concentration. Dexamethasone hemisuccinate loaded liposomes were successfully fabricated with a size of ~170 nm and low PDI (<0.1). Dexamethasone loaded liposomes were decorated with 5 mol% of a newly synthesized CPE terminating with an alkyl chain for anchoring to the liposome bilayer and 5% mPEG2 kDa-DSPE. The CPE decorated liposomes showed a positive zeta potential (+13 mV), while CPE/PEG coated liposomes displayed an almost neutral zeta potential of +1 mV due to the PEG shielding of the oligocationic CPE. No significant leakage of loaded drug and size change was found after surface decoration. Preliminary in vitro release studies in HEPES at pH 7.4 and 37 °C also demonstrated a slow release of dexamethasone hemi succinate for more than 20 days. In this study, dexamethasone liposomes were generated, and various formulation parameters were investigated. The liposome surface was engineered with a CPE/PEG coating to modulate residence time of liposomes in the vitreous. Further investigation with in vitro and in vivo models will provide information concerning drug delivery and the therapeutic profile of this system.

**Acknowledgments:** This project has received funding from the European Union’s Horizon 2020 research and innovation programme under the Marie Skłodowska-Curie grant agreement No 722717.

**References:** [1] Urtti, A. Challenges and obstacles of ocular pharmacokinetics and drug delivery. *Adv. Drug Deliv. Rev.* **2006**, *58*, 1131–1135. [2] Cuenca, N.; Fernández-Sánchez, L.; Campello, L.; Maneu, V.; De la Villa, P.; Lax, P.; Pinilla, I. Cellular responses following retinal injuries and therapeutic approaches for neurodegenerative diseases. *Prog. Retin. Eye Res.* **2014**, *43*, 17–75. [3] Cunha-Vaz, J.; Bernardes, R.; Lobo, C. Blood-retinal barrier. *Eur. J. Ophthalmol*. **2011**, *21*, S3–S9.

### 3.30. Microscopic Polymeric Depots for the Local Delivery of Anti-Inflammatory Molecules

Di FrancescoMartina^1^PrimaveraRosita^1^PalombaRoberto^1^BedingfieldSean K.^2^YuFang^2^DuvallCraig^2^Di MascoloDaniele^1^DecuzziPaolo^1^*1Laboratory of Nanotechnology for Precision Medicine, Italian Institute of Technology, 16163 Genova, Italy2Department of Biomedical Engineering, Vanderbilt University, Nashville, TN 37235, USA*Correspondence: paolo.decuzzi@iit.it

Local drug delivery represents an attractive alternative to systemic delivery in that it allows us to increase drug efficacy and selectivity at the target site while reducing toxicity in healthy tissues [1]. In general, larger particles achieve longer release profiles because they have the tendency to form a stable depot in the injection site and can store large amounts of therapeutic agents [2]. Here, a topdown approach is employed to realize microsized squared polymeric particles, called microplates (μPLs), for the local sustained release of anti-inflammatory drugs, such as Dexamethasone acetate (DEX). μPLs are polymeric particles made out of poly(lactic-co-glycolic acid (PLGA). Two different μPLs configurations were developed: one with an edge length of 20 μm and a height of 5 μm (Config. I) [3] and one with an edge length of 20 μm and a height of 10 μm (Config. II). During the synthesis process, different amounts of PLGA are used for the particle synthesis in order to modulate the Young’s modulus, without affecting the μPL geometry. Note that modulation of Young’s modulus allows us to match the properties of μPLs with the surrounding tissue, which is a fundamental feature in tissue depots. Config. I μPL s, realized with 5 mg of PLGA, show a stiffness value of about 2.08 ± 0.5 MPa, similar to that of cartilage tissue [4]. The anti-inflammatory activity of these particles is evaluated on RAW 264.7 cells and primary macrophages (BMDMs). Results show that DEX released from μPL s reduces the expression of the inflammatory cytokines on both cell lines at DEX concentration of 1 and 10 μM. The fine-tuning of the μPL geometry and polymer amounts provides the opportunity to modulate the diffusion of DEX molecules out of the μPL matrix, with continuous release for up to 60 days. A preliminary in vivo study was conducted. Saline buffer (control), free DEX, empty μPLs and DEX-μPLs (20 μg) were implanted into each mouse knee. Twenty-four hours post-injection, mice were subjected to cyclic mechanical loading at 9 N, 500 cycles, 5 times per week, for 4 weeks. Alexafluor-labeled collagen II-targeting monoclonal antibodies (mAbCII) were injected intravenously 72 h before sacrifice to quantify cartilage damage. IL-1β, IL-6, and TNF-α were assessed using TaqMan RT-PCR. Safranin-O and H&E staining were performed on the resulting histology, and scored by a blinded pathologist. Data will be soon available.

**Acknowledgments:** this project was partially supported by the European Research Council, under European Union’s Seventh Framework Programme (FP7/2007–2013)/ERC grant agreement no 616695, by the Italian Association for Cancer Research (AIRC) under the individual investigator grant No 17664, and by the European Union’s Horizon 2020 research and innovation Programme under the Marie Sklodowska-Curie grant agreement No 754490. The authors acknowledge the precious support provided by the Nikon Center, the Electron Microscopy and Nanofabrication facilities at the Italian Institute of Technology.

**References:** [1] Weiser, J.R.; Saltzman, W.M. Controlled release for local delivery of drugs: Barriers and models. *J. Control. Release* **2014**, 190, 664–673. [2] Kohane, D.S. Microparticles and nanoparticles for drug delivery. *Biotechnol. Bioeng.* **2007**, *96*, 203–209. [3] Di Francesco, M.; Primavera, R.; Romanelli, D.; Palomba, R.; Pereira, R.C.; Catelani, T.; Celia, C.; Di Marzio, L.; Fresta, M.; Di Mascolo, D.; et al. Hierarchical Microplates as Drug Depots with Controlled Geometry, Rigidity, and Therapeutic Efficacy. *ACS Appl. Mater. Interfaces* **2018**, *10*, 9280–9289. [4] Mansour, J.M. *Biomechanics of Cartilage. Kinesiology: The Mechanics and Pathomechanics of Human Movement*; Lippincott Williams and Wilkins: Philadelphia, PA, USA, 2003; pp. 66–79.

### 3.31. Spherical Polymeric Nanoparticles for the Delivery of Methotrexate to Atherosclerotic Plaques

Di FrancescoValentina^1^,^2^PalombaRoberto^2^FerreiraMiguel^2^ChiesaEnrica^3^GentaIda^3^ContiBice^3^,^4^DecuzziPaolo^2^*1Department of Informatics, Bioengineering, Robotics and System Engineering, University of Genoa, Via Opera Pia, 13, 16145 Genoa, Italy2Laboratory of Nanotechnology for Precision Medicine, Fondazione Istituto Italiano di Tecnologia, Via Morego, 30, 16163 Genoa, Italy3Department of Drug Sciences, University of Pavia, V.le Taramelli 12, 27100 Pavia, Italy4Polymerix s.r.l., V.le Taramelli 24, 27100 Pavia, Italy*Correspondence: paolo.decuzzi@iit.i

Atherosclerosis is a chronic inflammatory disease affecting the blood vessel walls. Its pathogenesis is based on the transformation of macrophages into foam cells, following the uptake of oxidized Low Density Lipoproteins (ox-LDL). Methotrexate (MTX) was recently proposed as a therapeutic molecule for the treatment of this pathology due to its anti-inflammatory activity. In this work, in order to reduce MTX toxicity and increase its half-life and overall therapeutic efficacy, MTX was bound to lipid chains, which were then used as constituents of Spherical Polymeric Nanoparticles (SPNs) [1]. SPNs were synthetized using two different approaches: a sonication-emulsion technique, and a microfluidic-assisted mixing (NanoAssemblr Benchtop instrument). Nanoparticles obtained with the two different fabrication approaches present comparable features. For the conventional sonication-emulsion approach, the SPN size is 205 nm with a PdI of 0.16, Zeta Pot is −45 mV; for the microfluidic mixing, the SPN size is 190 nm with a PdI of 0.2, Zeta Pot is −40 mV. In order to asses, the in vitro efficacy of the treatment with MTX-SPN, foam cells were first obtained, starting from primary rat Bone Marrow Derived Monocites (BMDM), upon treatment with ox-LDL. TEM and confocal imaging techniques were used to visualize ox-LDL accumulation into lysosomes. Ox-LDL trafficking was observed by time lapse microscopy. In order to visualize ox-LDL uptake, the molecule was conjugated to DIL. Then, the reversion of foam cells to normal macrophages was investigated upon treatment with MTX. Imaging analysis revealed a reduced amount of foam cells in the samples treated with MTX-SPNs. Genes expression analysis showed the down-regulation of CD36 and SRA-1 genes (foam cell markers) [2] and the up-regulation of the reverse cholesterol transporter (ABCA1) [3], proving the efficacy of the treatment. Inflammatory gene expression (IL-6, IL-1β and TNFα) on cells treated with MTX-SPNs showed a reduction in comparison to untreated foam cells. Cytotoxicity tests of MTX-SPNs were performed on BMDM, cells showed a good tolerance to the treatment at the used doses. In vivo experiments will be soon performed on atherosclerotic mice model to assess the reduction of the plaque of mice treated with MTX-SPNs.

**Acknowledgments:** This project was partially supported by the European Research Council, under the European Union’s Seventh Framework Program (FP7/2007–2013)/ERC grant agreement no. 616695, by the Italian Association for Cancer Research (AIRC) under the individual investigator grant no. 17664, and by the European Union’s Horizon 2020 research and innovation program under the Marie Skłodowska-Curie grant agreement No 754490. The authors acknowledge the precious support provided by the Nikon Center; the Electron Microscopy and Nanofabrication facilities at the Italian Institute of Technology.

**References:** [1] Stigliano, C.; Ramirez, M.R.; Singh, J.V.; Aryal, S.; Key, J.; Blanco, E.; Decuzzi, P. Methotraxate-Loaded Hybrid Nanoconstructs Target Vascular Lesions and Inhibit Atherosclerosis Progression in ApoE^−/−^ Mice. *Adv. Healthc. Mater.* **2017**, *6*, 1601286. [2] Rahaman, S.O.; Lennon, D.J.; Febbraio, M.; Podrez, E.A.; Hazen, S.L.; Silverstein, R.L. A CD36-dependent signaling cascade is necessary for macrophage foam cell formation. *Cell Metab.* **2006**, *4*, 211–221. [3] Coomes, E.; Chan, E.S.; Reiss, A.B. Methotrexate in atherogenesis and cholesterol metabolism. *Cholesterol* **2011**, *2011*, 503028.

### 3.32. Pullulan-Bioconjugates for Ocular Drug Delivery

KickovaEva^1^*BalassoAnna^1^MastrottoFrancesca^1^MostileElena^1^ZamborlinAgata^1^UrttiArto^2^SalmasoStefano^1^CalicetiPaolo^1^*1Department of Pharmaceutical and Pharmacological Sciences, University of Padova, Via F. Marzolo 5, 35131 Padova, Italy2University of Helsinki and University of Eastern Finland, 00014 Helsinki, Finland*Correspondence: eva.kickova@studenti.unipd.it (E.K.); paolo.caliceti@unipd.it (P.C.)

The anatomy of the eye is unique, and crossing the numerous physiologic barriers of the posterior eye segment to achieve an adequate drug concentration in the vitreous for therapeutic treatment is challenging [1,2]. Age-related macular degeneration (AMD), one of the most frequent diseases affecting the posterior eye segment, requires invasive techniques and repeated intravitreal injections for drug administration [3]. The aim of the present work is to generate a polysaccharide-drug conjugate for intravitreal administration that is endowed with prolonged residence time and drug release in the vitreous. Three Pullulan (Pull) derivatives have been generated as platforms to select strategies and releasable linkers for Rhodamine (Rh) conjugation through either an ether, hydrazone or an ester bond. The hydrophobic character of Rh and degree of conjugation 22% of glucopyranose unit (GPU) modification induced the self-assembly of the Pullulan derivatives into spherical particles with an average size of 25 nm. The Pull-et-Rh, Pull-hy-Rh and Pull-es-Rh derivative released 5, 50 and 75% of Rh respectively, over 10 days under conditions mimicking the vitreous. According to multiple tracking (MPT) analysis performed on ex-vivo porcine eye, the diffusivity in vitreous was found to be about 0.02 μm^2^/s for the three conjugates. Moreover, the cytotoxicity and uptake studies investigated on Retinal Pigment Epithelial cells (ARPE-19) confirmed the biocompatibility of the carrier and highlighted a strong cellular uptake. Based on the results, the hydrazone linker was selected for its sustained and prolonged release of Rh in comparison with the ester one. Dexamethasone, selected as a corticosteroid drug, was conjugated to a fluorescently-labeled Pullulan for the treatment of intraocular inflammation, which is generally associated with the majority of retinal eye diseases such as AMD. 0.42% Pullulan GPU were fluorescently-labeled with Cyanine3 and 0.33% were derivatized with Dexamethasone. The chemical identity of the conjugates was assessed by a combination of 1H NMR, 13C NMR, FT-IR, ESI-TOF, DLS, RP-HPLC and Elemental Analysis. Dedicated conditions are under exploration to improve the final conjugation yield of both Cyanine3 and Dexamethasone and to assess the colloidal behavior of the resulting supramolecular system. We have selected the synthetic strategy to load the Pullulan based carrier with drugs and elucidated the colloidal features of the resulting nanocarrier. The carrier undergoes a very slow diffusivity in the vitreous that may extend its residence time, and thus, the level of local drug release. The hydrazone-containing linker was shown to be the most suitable for prolonged intraocular drug release. In vitro and ex vivo studies with Dexamethasone loaded Pullulan will elucidate the biopharmaceutical behavior of this class of the supramolecular drug vehicle.

**Acknowledgments:** This project has received funding from the European Union’s Horizon 2020 research and innovation program under the Marie Skłodowska-Curie grant agreement N° 722717.

**References:** [1] Del Amo, E.M.; Rimpelä, A.K.; Heikkinen, E.; Kari, O.K.; Ramsay, E.; Lajunen, T.; Schmitt, M.; Pelkonen, L.; Bhattacharya, M.; Richardson, D.; et al. Pharmacokinetic aspects of retinal drug delivery. *Prog. Retin. Eye Res.* **2017**, *57*, 134–185; [2] Peynshaert, K.; Devoldere, J.; de Smedt, S.C.; Remaut, K. In vitro and ex vivo models to study drug delivery barriers in the posterior segment of the eye. *Adv. Drug Deliv. Rev.* **2018**, *126*, 44–57. [3] Bansal, P.; Garg, S.; Sharma, Y.; Venkatesh, P. Posterior segment drug delivery devices: Current and novel therapies in development. *J. Ocul. Pharmacol. Ther.* **2016**, *32*, 135–144.

### 3.33. A Tissue microChamber for the Mass Transport Analysis of Molecular and Nano-Medicines

LusiV.^1^MizrahyS.^1^,^2^,^3^,^4^FerreiraM.^1^CocliteA.^1^PereiraR.C.^1^PeerD.^2^,^3^,^4^DecuzziP.^1^*1Laboratory of Nanotechnology for Precision Medicine, Fondazione Istituto Italiano di Tecnologia, Via Morego 30, 16163 Genoa, Italy2Laboratory of Precision NanoMedicine, School of Molecular Cell Biology and Biotechnology, George S. Wise Faculty of Life Science; Department of Materials Sciences and Engineering, Iby and Aladar Fleischman Faculty of Engineering, Tel Aviv University, Tel Aviv 6997801, Israel3Center for Nanoscience and Nanotechnology, Tel Aviv University, Tel Aviv 6997801, Israel4Cancer Biology Research Center, Tel Aviv University, Tel Aviv 6997801, Israel*Correspondence: paolo.decuzzi@iit.it

The transport of drug molecules and nanomedicines (NMs) within any biological tissue depends on their size, surface electrostatic charge, composition, mechanical properties as well as on the architecture and porosity of the host tissue. NMs features can be tailored during the fabrication process whereby the size, shape, surface and mechanical properties are precisely controlled over multiple scales [1,2]. In this work, a ‘*Tissue microChamber*’ was developed in order to reproduce ex-vivo a biological tissue and investigate the mass transport of drugs and nanomedicines. Specifically, a three-dimensional collagen hydrogel was placed in a microfabricated chamber and a needle was used to deploy, under controlled conditions, a fluorescent working fluid within the hydrogel itself. By detecting via fluorescent microscopy the spreading over time of the working fluid and its particulate content, the diffusion coefficients of drug molecules and NMs was estimated. First, the case of dextran molecules with three different molecular weights (4, 40, 250 kDa) was considered. Then, the transport analysis was extended to different nanomedicines. Specifically, 200 nm of commercially-available polystyrene carboxylate beads, ~200 nm spherical polymeric nanoparticles (SPNs), prepared from PLGA core coated by a PEG monolayer [3], and ~200 lipid nanoparticles coated with two different molecular weights hyaluronic acid (5kDa-HA and 700 kDa-HA) layer [4] were considered as representative nanomedicines. Through Mean Square Displacement (MSD) analyses, the diffusion coefficient of these particulate systems was quantified. Results showed that a higher percentage of PEGylation on their surfaces increases the diffusion coefficient, perhaps by exerting a lubricating action on the surrounding collagen tissue, whereas high molecular weight HA chains tend to more efficiently entangle with the fibrous structure of the collagen tissue, thereby reducing particle mobility. Taken together, these data provide useful information for optimizing the design of nanomedicines in order to improve spreading within the target biological tissue.

**Acknowledgments:** This project was partially funded by the European Union’s Seventh Framework Programme (FP7/2007–2013)/ERC grant agreement No 616695, by the Italian Association for Cancer Research (AIRC) under the individual investigator grant No 17664, and by the European Union’s Horizon 2020 research and innovation programme under the Marie Sklodowska-Curie grant agreement No 754490.

**References:** [1] Palomba, R.; Palange, A.L.; Rizzuti, I.F.; Ferreira, M.; Cervadoro, A.; Barbato, M.G.; Canale, C.; Decuzzi, P. Modulating Phagocytic Cell Sequestration by Tailoring Nanoconstruct Softness. *ACS Nano* **2018**, *12*, 1433–1444. [2] Di Francesco, M.; Primavera, R.; Romanelli, D.; Palomba, R.; Pereira, R.C.; Catelani, T.; Celia, C.; Di Marzio, L.; Fresta, M.; Di Mascolo, D.; et al. Hierarchical Microplates as Drug Depots with Controlled Geometry, Rigidity, and Therapeutic Efficacy. *ACS Appl. Mater. Interfaces* **2018**, *10*, 9280–9289. [3] Aryal, S.; Key, J.; Stigliano, C.; Landis, M.D.; Lee, D.Y.; Decuzzi, P. Positron emitting magnetic nanoconstructs for PET/MR imaging. *Small* **2014**, *10*, 2688–2696. [4] Mizrahy, S.; Goldsmith, M.; Leviatan-Ben-Arye, S.; Kisin-Finfer, E.; Redy, O.; Srinivasan, S.; Shabat, D.; Godin, B.; Peer, D. Tumor targeting profiling of hyaluronan-coated lipid based-nanoparticles. *Nanoscale* **2014**, *6*, 3742–3752.

### 3.34. HpβCD Nanogels as Ibuprofen Transdermal Delivery Systems

MenniniNatascia*CirriMarziaMaestrelliFrancescaMuraPaolaDepartment of Chemistry, University of Florence,50019 Florence, Italy*Correspondence: natascia.mennini@unifi.it

Ibuprofen [2-(4-isobutylphenyl) propionic acid] is a non-steroidal anti-inflammatory drug (NSAID) which is poorly soluble in water, and used to relieve inflammation, swelling, stiffness and joint pain. However, the frequent oral use of this drug can cause several side effects, including decreased gastric cytoprotection, impairment of renal function, and inhibition of platelet aggregation. Therefore, the development of topical and/or transdermal dosage forms making it possible to both avoid the gastrointestinal side-effects of the oral route and to provide consistent drug levels at the application site for prolonged times is considered very desirable, particularly in the treatment of local inflammatory pain, like muscle aches and arthritis. However the development of effective ibuprofen topical delivery systems is still a challenge, due to its intrinsically poor skin permeability. The development of suitable nanometric systems, able to encapsulate the drug in colloidal structures and enhance its skin permeability, could be an effective strategy to improve ibuprofen topical bioavailability. With this aim, in the present study, a cyclodextrin-based nanogel (CD-nanogel) was developed by combining the advantages of 2-hydroxylpropyl-β-cyclodextrin, as both solubilizing agent and transdermal permeation enhancer [1], with the nanogels properties, that include the ability to regulate and prolong drug release and facilitate the crossing of tissue barriers [2]. The CD-nanogel formulation was prepared by a W/O emulsion-solvent evaporation technique with simultaneous crosslinking [2], followed by a hot/cold dispersion method [3] using ethylene glycol diglycidyl ether (EGDE) and hydroxypropyl methylcellulose (HPMC) respectively as cross-linking and gelling agent. Between these two steps the drug loading process was carried out. Conventional HPMC gels, and HPMC gels containing HPßCD were also prepared for comparison purposes to evaluate the role of the nanogel. The obtained gels were characterized for drug loading efficiency and rheological properties. In vitro skin permeation experiments performed by Franz diffusion cells using a cellulose nitrate artificial membrane impregnated with lauryl alcohol as lipid phase simulating the epidermal barrier [4] demonstrated the effective ability of the developed CD-nanogel to promote a sustained release of the drug, and to increase its transdermal permeability, when compared with a corresponding HPMC gel prepared without cyclodextrin.

**References:** [1] Kurkov, S.K.; Loftsson, T. Cyclodextrins. *Int. J. Pharm.* **2013**, *453*, 167–180. [2] Moya-Ortega, M.D.; Alvarez-Lorenzo, C.; Sigurdsson, H.H.; Concheiro, A.; Loftsson, T. Cross-linked hydroxypropyl-β-cyclodextrin and γ-cyclodextrin nanogels for drug delivery: Physicochemical and loading/release properties. *Carbohydr Polym.* **2012**, *87*, 2344–2351. [3] Turowski, M.; Deshmukh, B.; Harfmann, R.; Conklin, J.; Lynch, S. A method for determination of soluble dietary fiber in methylcel luloseand hydroxypropyl methylcel lulose food gum. *J. Food Comp. Anal.* **2007**, *20*, 420–429. [4] Mura, P.; Bragagni, M.; Mennini, N.; Cirri, M.; Maestrelli, F. Development of liposomal and microemulsion formulations for transdermal delivery of clonazepam: Effect of randomly methylated β-cyclodextrin. *Int. J. Pharm.* **2014**, *475*, 306–314.

### 3.35. Hybrid Self-Assembling Nanoparticles: Tuning Lipid Composition for Enhanced miRNA Delivery

ScottiLorena^1^CampaniVirginia^1^ZappavignaSilvia^2^AbateMarianna^2^PorruManuela^3^LeonettiCarlo^3^CaragliaMichele^2^De RosaGiuseppe^1^*1Department of Pharmacy, University of Studies of Naples Federico II, Naples 80131, Italy2Department of Biochemistry, Biophysics and General Pathology, University of Campania “Luigi Vanvitelli”, 80131 Napoli, Italy3Department of Research, Diagnosis and Innovative Technologies, Regina Elena National Cancer Institute, 00144 Roma, Italy*Correspondence: gderosa@unina.it

MicroRNA (miRNA) are non-coding RNAs involved in regulation of gene expression and potential therapeutic agents in the treatment of diseases. However, the therapeutic use of miRNA is hampered by biopharmaceutical issues, requiring the use of ad hoc developed delivery systems [1]. Previously, hybrid self-assembling nanoparticles (SANPs) for the delivery of anionic drugs, e.g., bisphosphonates, have been developed [2] and successfully used in different types of tumors, such as glioblastoma (GBM). Here, we propose SANPs to deliver miRNA. To do this, SANP lipid composition was optimized in terms of cationic lipids, the presence of neutral lipids, and type of PEGylated lipids. As model miRNA, miR603, potentially useful to restore chemoresistance in GBM, was used. All formulations were characterized in terms of mean diameter, polydispersity index, zeta potential, miRNA encapsulation, NPs stability in BSA, serum and red blood cells, NPs hemolytic activity. Moreover, the SANPs were tested in vitro (cytotoxicity, uptake) on two different cell lines of GBM namely U87MG and LN229. The majority of the formulations showed a mean diameter lower 170 nm and a polydispersity index lower than 0.2. The zeta potential was positive (between +10 mV and +45 mV) and the miRNA encapsulation efficiency was between 75% and 100%. The formulations were stable in BSA and serum four hours. Finally, the majority of the formulations showed a hemolysis lower than 2%. In vitro studies on two different cell lines served to highlight the formulations that were no toxic. Such formulations were used in the following miRNA intracellular uptake and cell viability studies. The uptake studied showed that the miR603 incapsulated in SANPs leads to better miR603 delivery than in the case of other transfection agents, e.g., lipofectamine. Biodistribution SANPs encapsulating miRNAs, following intravenous administration in an animal model of GBM, are ongoing, and will provide useful information on the SNAP accumulation in different organs and in the tumor. If successful, this approach will be worthy of deeper study, e.g., activity studies, and of being extended to other tumors in which the delivery of ncRNAs (miRNA mimics, antagomir, small interfering RNA) could be beneficial.

**Acknowledgments:** The project was kindly supported by Phospholipids Research Center, Heidelberg, Germany.

**References:** [1] Li, Z.; Rana, T.M. Therapeutic targeting of microRNAs: Current status and future challenges. *Nat. Rev. Drug Discov.* **2014**, *13*, 622–638. [2] Salzano, G.; Zappavigna, S.; Luce, A.; D’Onofrio, N.; Balestrieri, M.L.; Grimaldi, A.; Lusa, S.; Ingrosso, D.; Artuso, S.; Porru, M. Transferrin-targeted nanoparticles containing zoledronic acid as a potential tool to inhibit glioblastoma growth. *J. Biomed. Nanotechnol.* **2016**, *12*, 811–830.

### 3.36. Non-Viral Gene Therapy for Corneal Inflammation

Vicente-PascualMónica^1^*Rodríguez-CastejónJulen^1^del Pozo-RodríguezAna^1^SolinísM. Ángeles^1^MuntoniE.^2^FogliettaF.^2^Rodríguez-GascónAlicia^1^BattagliaL.S.^2^1Pharmacokinetic, Nanotechnology and Gene Therapy Group (PharmaNanoGene), Faculty of Pharmacy, Centro de Investigación Lascaray Ikergunea, UPV/EHU, 01006 Vitoria-Gasteiz, Spain2Università degli Studi di Torino, Dipartimento di Scienza e Tecnologia del Farmaco, 10125 Torino, Italy*Correspondence: monica.vicente@ehu.eus

Corneal inflammation is the underlying process of many eye diseases. Interleukin-10 (IL-10) administration has been proposed as an efficient treatment for inflammation, and gene therapy could provide a sustained IL-10 production in corneal cells, and therefore, have a long-term anti-inflammatory effect [1]. The aim of this study was to develop an inflammation model in Human Corneal Epithelial (HCE) cells, and to evaluate the efficacy of two non-viral transfection systems based on Solid Lipid Nanoparticles (SLN) against corneal inflammation. Inflammation model in HCE cells: different methodological conditions, as composition of the culture medium or TNF-α concentrations, were assayed. Final conditions were: culture of the cells during 24 h with complete medium (CM), addition of medium without supplements (MWS) and incubation overnight, 24 h of incubation with a mixture of TNFα (10 ng/mL) with MWS (controls just with MWS) [2]. After this step, cells were left for 1 h with CM before transfection. The vectors were removed 4 h later. Quantification of secreted IL-6 and IL10 was performed by ELISA. Detection of CD44 receptor was performed by Inmunocytochemistry [4]. Preparation of SLNs and vectors: The vectors (HA-SLN and DX-SLN) were prepared with the plasmid, SLN, protamine (P), and hyaluronic acid (HA) or dextran (DX), respectively, as previously described [3]. The vectors were characterized in a Zetasizer Nano series-Nano ZS. Percentage of transfected cells and cell viability were measured at 72h using a CytoFLEX flow cytometer. The protocol selected was able to induce the inflammation of HCE cells; the values of the secreted IL6 increased in inflamed cells up to 0.058 pg/mL/n° cells at 72 h (fivefold higher than in control cells). The upregulation of CD44 receptor confirmed the inflammation of HCE cells, as can be seen in Figure 1C. The particle size of the vectors was in the nanometer range (HA-SLN: 331 ± 7.5 nm, DX-SLN: 199.8 ± 0.4 nm) and their surface charge was positive (HA-SLN: +30.3 ± 1.8 mV, DX-SLN: +38.4 ± 1.6 mV). The percentage of transfection was 8% and 6% for HA-SLN and DX-SLN, respectively, in both control and inflamed cells. Both vectors were able to induce the production of IL-10, although HA-SLN to a higher extent. This vector, which has previously shown the capacity to transfect rabbit cornea explants [1], presents high affinity for the CD44 receptor [4], which is overexpressed in inflamed HCE cells. Cell viability was always higher than 90%. In conclusion, this work shows the capacity of these vectors to induce the production of IL10 in inflamed corneal cells, and therefore, their potential utility for the treatment of inflammatory corneal diseases.

**Acknowledgments:** This work was supported by the Spanish Ministerio de Economía y Competitividad (SAF2014-53092-R) and by the UPV/EHU (PPG17/65, GIU17/32).

**References:** [1] Vicente-Pascual, M.; Albano, A.; Solinís, M.Á.; Serpe, L.; Rodríguez-Gascón, A.; Foglietta, F.; Muntoni, E.; Torrecilla, J.; Pozo-Rodríguez, A.D.; Battaglia, L. Gene delivery in the cornea: In vitro & ex vivo evaluation of solid lipid nanoparticle-based vectors. *Nanomedicine* **2018**, *13*, 1847–1854. [2] Soriano-Romaní, L.; Vicario-de-la-Torre, M.; Crespo-Moral, M.; López-García, A.; Herrero-Vanrell, R.; Molina-Martínez, I.T.; Diebold, Y. Novel anti-inflammatory liposomal formulation for the pre-ocular tear film: In vitro and ex vivo functionality studies in corneal epithelial cells. *Exp. Eye Res.* **2017**, *154*, 79–87. [3] Apaolaza, P.S.; Del Pozo-Rodríguez, A.; Torrecilla, J.; Rodríguez-Gascón, A.; Rodríguez, J.M.; Friedrich, U.; Weber, B.H.; Solinís, M.A. Solid lipid nanoparticle-based vectors intended for the treatment of X-linked juvenile retinoschisis by gene therapy: In vivo approaches in Rs1h-deficient mouse model. *J. Control. Release* **2015**, *217*, 273–283. [4] Apaolaza, P.S.; Delgado, D.; del Pozo-Rodríguez, A.; Gascón, A.R.; Solinís, M.Á. A novel gene therapy vector based on hyaluronic acid and solid lipid nanoparticles for ocular diseases. *Int. J. Pharm.* **2014**, *465*, 413–426.

## 4. Conclusions

The 2018, CRS Italy Chapter annual workshop pursued the idea of allowing our community to meet, sharing scientific results and experiences, bringing many opportunities for people to mingle and discuss collaborations and future research. Special attention was paid to young researchers; among the aims of our association, three “best PhD thesis” prizes were awarded. This year, the recipients were: Dr. Chiara Bastiancich (Université Catholique de Louvain, Belgium), Dr. Patrizia Nadia Hanieh (Sapienza University of Roma, Italy) and Dr. Katia Maso (University of Padua, Italy). Also, three young researchers were awarded the “best poster”: Dr. Umberto Musazzi, Dr. Silvia Franzè and Dr. Chiara Bastiancich.

We will try to continue organizing such exciting workshops in the future, and will work to bring more attendees from abroad to improve networking among people involved in drug delivery technologies.

## Figures and Tables

**Figure 1:**
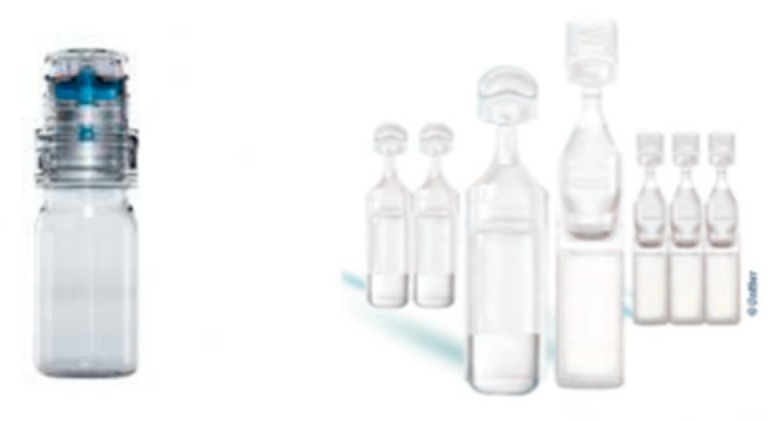
Preservative free multidose and BFS monodoses.

**Figure 1:**
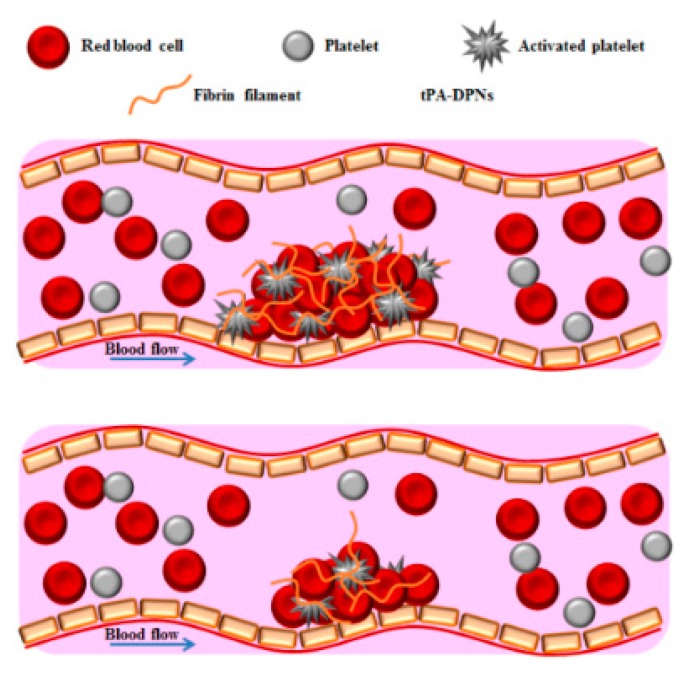
Schematic representation of the action of tPA-DPNs on a blood clot.

**Figure 1:**
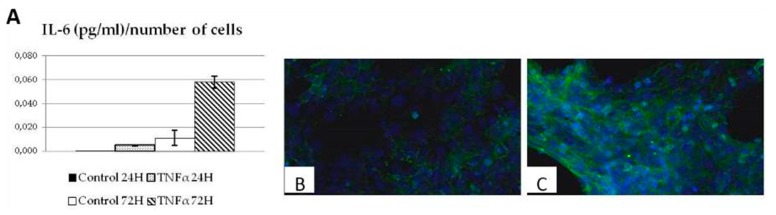
(**A**) Quantification of IL-6; (**B**) CD44 receptor in control; and (**C**) in inflamed HCE cells.

